# Deciphering the Non-Equivalence of Serine and Threonine *O*-Glycosylation Points: Implications for Molecular Recognition of the Tn Antigen by an anti-MUC1 Antibody**

**DOI:** 10.1002/anie.201502813

**Published:** 2015-06-26

**Authors:** Nuria Martínez-Sáez, Jorge Castro-López, Jessika Valero-González, David Madariaga, Ismael Compañón, Víctor J Somovilla, Míriam Salvadó, Juan L Asensio, Jesús Jiménez-Barbero, Alberto Avenoza, Jesús H Busto, Gonçalo J L Bernardes, Jesús M Peregrina, Ramón Hurtado-Guerrero, Francisco Corzana

**Affiliations:** Departamento de Química, Universidad de La Rioja, Centro de Investigación en Síntesis Química 26006 Logroño (Spain) E-mail: jesusmanuel.peregrina@unirioja.es francisco.corzana@unirioja.es; Department of Chemistry, University of Cambridge Lensfield Road, Cambridge CB2 1EW (UK); Institute of Biocomputation and Physics of Complex Systems (BIFI), University of Zaragoza, BIFI-IQFR (CSIC) Joint Unit Edificio I+D, 50018 Zaragoza (Spain) Fundación ARAID, Edificio Pignatelli 36 Zaragoza (Spain); Departament de Química Analítica i Química Orgànica, Universitat Rovira i Virgili C/Marcellí Domingo s/n, 43007 Tarragona (Spain); Instituto de Química Orgánica General, IQOG-CSIC, Juan de la Cierva 3 28006 Madrid (Spain); Structural Biology Unit, CIC bioGUNE, Parque Tecnológico de Bizkaia Building 801 A 48160 Derio (Spain) IKERBASQUE, Basque Foundation for Science 48011 Bilbao (Spain) Department of Chemical and Physical Biology, Centro de Investigaciones Biológicas CSIC, Ramiro de Maeztu 9, 28040 Madrid (Spain); Instituto de Medicina Molecular, Faculdade de Medicina da Universidade de Lisboa 1649-028 Lisboa (Portugal)

**Keywords:** antibodies, conformation analysis, glycopeptides, molecular recognition, X-ray diffraction

## Abstract

The structural features of MUC1-like glycopeptides bearing the Tn antigen (α-*O*-GalNAc-Ser/Thr) in complex with an anti MUC-1 antibody are reported at atomic resolution. For the α-*O*-GalNAc-Ser derivative, the glycosidic linkage adopts a high-energy conformation, barely populated in the free state. This unusual structure (also observed in an α-*S*-GalNAc-Cys mimic) is stabilized by hydrogen bonds between the peptidic fragment and the sugar. The selection of a particular peptide structure by the antibody is thus propagated to the carbohydrate through carbohydrate/peptide contacts, which force a change in the orientation of the sugar moiety. This seems to be unfeasible in the α-*O*-GalNAc-Thr glycopeptide owing to the more limited flexibility of the side chain imposed by the methyl group. Our data demonstrate the non-equivalence of Ser and Thr *O*-glycosylation points in molecular recognition processes. These features provide insight into the occurrence in nature of the APDTRP epitope for anti-MUC1 antibodies.

The Tn antigen (α-*O*-GalNAc-Ser/Thr) is one of the most specific human tumor-associated structures.[1] This entity, which is also implicated in HIV infection,[2] is expressed in approximately 90 % of carcinomas, and a direct correlation between the aggressiveness of the carcinoma and the occurrence of the antigen has been observed.[3] Consequently, the Tn antigen has found widespread use as biomarker and as a potential therapeutic target against cancer.[1, 4–7] Structural analysis of Tn antigen bound to its biological targets is thus of great significance for elucidating the mechanism of recognition, as well as for engineering novel antibodies and biosensors. In general, the Tn antigen is referred to as *N*-acetylgalactosamine (GalNAc) α-*O*-linked to serine (Ser) or threonine (Thr), without specifying which of the two amino acids the GalNAc is linked to. However, we and others have observed the existence of subtly different conformational behaviors in solution of the basic Ser- and Thr-containing structures.[8–14]

Herein, we present a detailed analysis of the interaction of these two Tn determinants, as MUC1 glycopeptides, to an anti-MUC1 antibody. MUC1 is a heavily *O*-glycosylated membrane glycoprotein consisting of tandem repeats of 20 amino acids (AHGVTSAPDTRPAPGSTAPP), with five possible glycosylation sites.[15, 16] This protein is overexpressed and partially glycosylated in cancer cells. Consequently, some peptide fragments that are masked in healthy cells, such as APDTRP and their glycosylated analogues, are now accessible and can interact with the immune system. Although the observed enhancement of antibody affinity has been attributed to conformational changes induced by the glycan in the peptide backbone,[17–20] the molecular basis for this observation remains unclear.

To our knowledge, the only reported crystal structure of a complex between an antibody and a GalNAc-containing glycopeptide is unrelated to mucins.[21] In addition, the X-ray structure of a model anti-MUC1 antibody (SM3) in complex with a mucin is limited to a naked peptide.[22] Moreover, an NMR study on this peptide and its corresponding GalNAc-glycopeptide bound to SM3 led to the hypothesis that the sugar residue fixes the bioactive conformation of the peptide fragment and interacts via the *N*-acetyl group with the surface of the antibody.[23] However, no detailed information on the intermolecular interactions could be deduced from this ligand-based NMR analysis.

A detailed analysis of the interactions between the SM3 antibody and two synthetic glycopeptides bearing the α-*O*-GalNAc-Thr and α-*O*-GalNAc-Ser antigens (m1* and m2* respectively; Figure 1) is presented herein. These molecules include the tandem repeat sequence of MUC1. The SM3 antibody was selected because its epitope recognition mode is similar to that of other anti-MUC1 antibodies,[24] which expands the scope of these results, and also because of its potential for use in the early diagnosis and treatment of breast cancer.[22]

**Figure 1:**
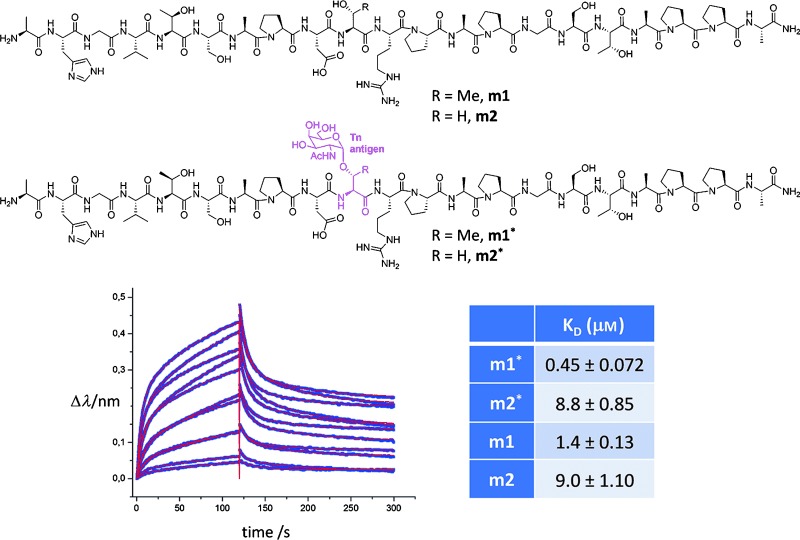
The MUC1-like peptides and glycopeptides studied in this work (upper panel). Bio-layer interferometry (BLI) curves and fit obtained for glycopeptide m1* and scFv-SM3, together with the *K*_D_ constants derived from BLI experiments for all of the MUC1-related compounds (lower panel).

The influence of the chemical nature of the underlying amino acid, as well as the GalNAcylation, on antibody binding affinity was first evaluated. For this purpose, the related naked peptides (**m1** and **m2**, respectively; Figure 1) were also synthesized and tested. The *K*_D_ constants for the MUC1 variants with the scFv-SM3 antibody were experimentally determined through bio-layer interferometry (BLI) experiments. The higher affinity (around 3-fold) of SM3 for glycosylated m1* compared the naked peptide **m1** was confirmed by these tests[24] (Figure 1). Furthermore, the Ser-containing compounds showed significantly lower affinity, thus highlighting the differences between the two Tn antigens. These results were corroborated by ELISA tests (see the Supporting Information).

To explain these results at the atomic level, a scFv-SM3 antibody was produced and purified. High quality crystals of the SM3:**1**, SM3:1*, and SM3:2* complexes were obtained, where **1**, 1* and 2* are simplified models of **m1**, m1* and m2*, respectively (Figure 2 a, PDB IDs 5a2j, 5a2k, and 5a2i). These compounds include the peptide fragment that represents the minimal epitope recognized by most anti-MUC1 antibodies.[24]

**Figure 2:**
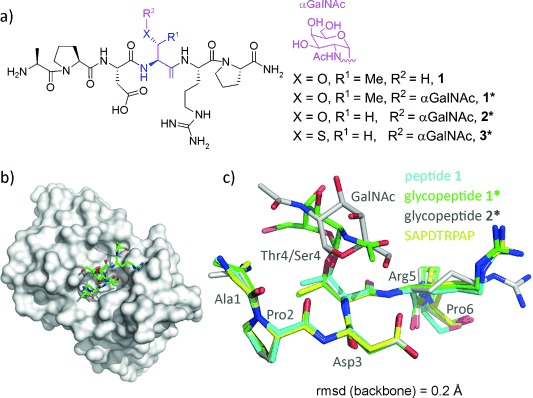
a) Simplified MUC1 variants used for crystallization. b) Surface representation of SM3 in complex with 1*. The antigen is shown as a stick model with carbon atoms in green. c) Superposition of the peptide backbone of compounds 1, 1*, 2*, and the SAPDTRPAP peptide[22] in complex with SM3.

The obtained crystals enabled solution of the structures at high resolution (<2.0 Å, see the Supporting Information). Crystallographic analysis revealed that the surface groove of the recombinant SM3 antibody nicely fits all of the peptide residues in the three studied complexes (Figure 2 b), independent of the presence of the sugar moiety. Moreover, the overall conformation of the peptide fragment of the different simplified MUC1 variants is nearly identical and is similar to that found in the crystal structure reported for the naked peptide[22] (Figure 2 c and Figure 3).

**Figure 3:**
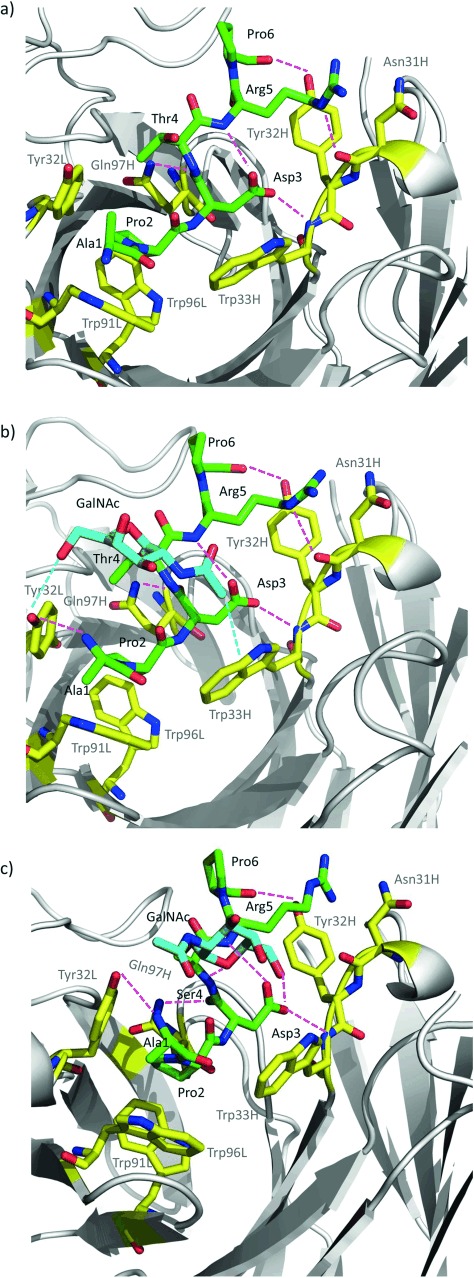
Key binding interactions of peptide 1 (a), glycopeptide 1* (b), and glycopeptide 2* (c) with SM3 mAb, as observed in the X-ray crystal structures. Peptide and glycopeptide carbon atoms are shown in green. GalNAc carbon atoms are shown in cyan. Carbon atoms of key residues of SM3 are colored yellow. Blue dashed lines indicate interactions between GalNAc and SM3 surface, and pink dashed lines indicate hydrogen bonds between peptide backbones and SM3 antibody.

Therefore, the presence of the GalNAc moiety, regardless of the attached amino acid (Ser or Thr), does not significantly affect the conformation of the peptide backbone in the SM3-bound state. In fact, the antibody–antigen pairs display the same pattern of interactions with each of the three substrates (**1**, 1* and 2*) and with the reported peptide[22] (Figure 3). The stabilizing contacts in these complexes involve several hydrogen bonds, some of them mediated by water molecules, as well as several stacking interactions (Figure 3 and the Supporting Information). Pro2 stacks with Trp91L, Trp96L, and Tyr32L, while the side-chains of Asp3 and Arg5 are engaged in hydrophobic contacts with Trp 33H and Tyr 32H, respectively. Additionally, in compounds 1* and 2*, the NH group of Ala1 and the carbonyl group of Thr4/Ser4 are involved in hydrogen bonds with Tyr 32L and Gln97H, respectively. The 3D presentation of the side chain of Arg5 in glycopeptide 2* (Figure 2 c) differs slightly from that found for the other analogues. As a result, there is no hydrogen bond between the side chain of Arg5 and the carbonyl group of Asn31H. This interaction, among other factors (see below), may be at the cause of the low affinity of SM3 for 2*.

Interestingly, the major difference between glycopeptides 1* and 2* bound to SM3 resides in the geometry of the glycosidic linkage (Figure 4 a). In fact, in the SM3:1* complex, this linkage adopts the expected *exo*-anomeric/syn conformation, with *ϕ* and *ψ* values of around 63° and 91°, respectively.[9] This conformation is similar to that found for a non-related MUC1 glycopeptide bound to 237-mAb[21] (Figure 4 b) and that exhibited for 1* bound to a model lectin (Soybean agglutinin).[25] This geometry allows the formation of an intermolecular hydrogen bond between the hydroxymethyl group of GalNAc and the side chain of Tyr32L on the SM3 antibody. Moreover, the *N*-acetyl group of the sugar stacks with the aromatic ring of Trp 33H, thus providing the impetus for the observed selectivity of SM3 for GalNAc-containing antigens. This interaction is compatible with the previous solution NMR data.[23] By contrast, the GalNAc unit of compound 2* establishes only weak water-mediated hydrogen bonds with the antibody (see the Supporting Information). The glycosidic linkage shows a high-energy conformation, with a *ψ* value of around −97° (Figure 4 a, c). This conformation is barely populated for the α-*O*-GalNAc-Ser motif in solution. In fact, the conformational analysis performed on glycopeptides 1* and 2* in the free state in water by means of NMR experiments and Molecular Dynamics (MD) simulations with time-averaged restraints[9] indicates that compound 2* displays this conformation with a population approximately 20 % (see the Supporting Information). As previously observed by us,[8, 9] while in compound 1*, the glycosidic linkage adopts mainly the typical eclipsed conformation, in variant 2* it prefers to adopt the alternate conformation. According to the X-ray structure, the unusual conformation of the glycosidic linkage in compound 2* bound to the antibody is stabilized by two intramolecular hydrogen bonds with the peptide chain (Figure 5). The GalNAc endocyclic oxygen O5 engages in a hydrogen bond with the NH group of the attached Ser and the O6 interacts with the side chain of Asp3. To our knowledge, this geometry of the glycosidic linkage has not been previously observed for protein-bound Tn-containing peptides. It is important to note that the quality of the SM3:2* structure was lower and that the B-factor for 2* was higher compared to the other structures, which may indicate some degree of flexibility.

**Figure 4:**
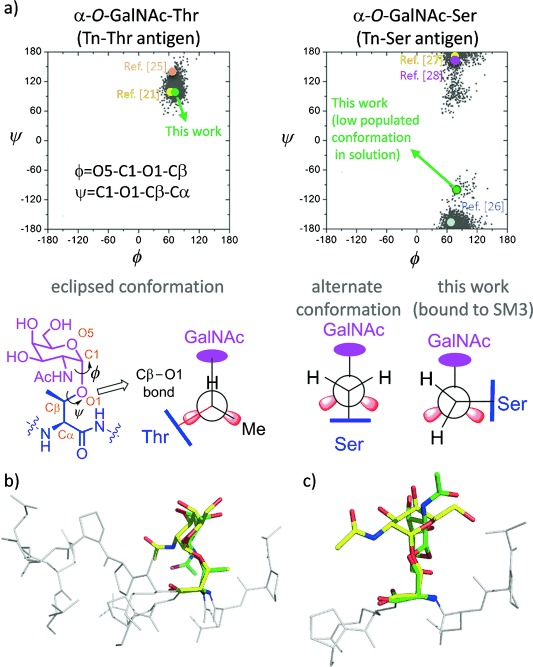
a) Distribution of *ϕ*/*ψ* torsional angles found for α-*O*-GalNAc-Ser and α-*O*-GalNAc-Thr in water,[8, 9] together with the geometries found in X-ray structures for these determinants when bound to some biological targets.[21, 25–28] Newman projections of Cβ–O1 bond are shown. b) Superposition of the Tn antigen moiety α-*O*-GalNAc-Thr bound to SM3 (in green) and 237-mAb (in yellow; mAb=monoclonal antibody).[21] c) Superposition of the Tn antigen moiety α-*O*-GalNAc-Ser bound to SM3 mAb (in green) and to HPA lectin (in yellow).[27]

**Figure 5:**
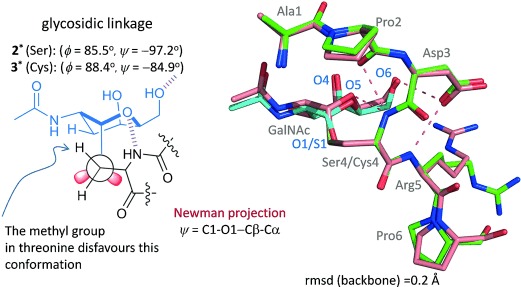
Conformation of glycopeptides 2* (in green and cyan) and 3* (in brown) in complex with scFv-SM3 antibody, together with the geometry of the glycosidic linkage and the hydrogen bonds established with the peptide fragment.

MD simulations performed for the SM3:2* complex corroborated this flexibility in the bound state. The X-ray conformation was retained only for the first 4 ns of the trajectory (see the Supporting Information). Then, the glycosidic linkage exhibits the typical parallel orientation found for this compound in the free state.[8] To reinforce the significance of the β-methyl group of the threonine residue for the conformation of the glycosidic linkage, and to corroborate the exceptional 3D conformation of glycopeptide 2* in the complex with the antibody, we solved the structure of the cysteine analogue in complex with scFv-SM3 (compound 3* in Figure 2, PDB ID: 5a2L). Notably, the GalNAc unit and the peptide backbone adopt an almost identical spatial conformation in glycopeptides 2* and 3*, with the main difference being the conformation of the side chain of Arg5 (Figure 5). Therefore, the selection of a particular peptide structure by the antibody is somehow propagated to the carbohydrate through specific carbohydrate/peptide contacts, thereby forcing a drastic change in the orientation of the sugar moiety. This situation is not possible in the Thr-containing derivative owing to the limited conformational freedom of its side chain imposed by the methyl group (Figure 5). As a result, the GalNAc unit adopts a completely different presentation when linked to the threonine residue, with most of the hydroxy groups exposed and able to interact with the corresponding partners of the immune system.

In conclusion, we have uncovered, at the atomic level, the reasons why Ser- and Thr-linked glycopeptides bind differently to SM3. We have provided experimental evidences for distinct presentations of the Tn-carrying serine (α-GalNAc-Ser) and threonine (α-GalNAc-Thr) antigens when bound to SM3. The reasons for the observed limited enhancement in SM3 affinity in 1* versus **1** can be attributed to the weak hydrogen bond between O6 and Tyr32L, and a hydrophobic contact between the methyl group of the GalNAc unit and Trp33H. These findings emphasize the differences between these two Tn antigens in the context of recognition by anti-MUC1 antibodies and may have important implications for the design of novel antibodies and biosensors. In addition, our findings may provide insight into the occurrence in nature of the APDTRP epitope for anti-MUC1 antibodies.

## Supporting Information

As a service to our authors and readers, this journal provides supporting information supplied by the authors. Such materials are peer reviewed and may be re-organized for online delivery, but are not copy-edited or typeset. Technical support issues arising from supporting information (other than missing files) should be addressed to the authors.

miscellaneous_information
