# Supplementary material for: Deciphering the Non-Equivalence of Serine and Threonine O-Glycosylation Points: Implications for Molecular Recognition of the Tn Antigen by an anti-MUC1 Antibody
Source: Angew Chem Int Ed Engl. 2015 Jun 26;54(34):9830–4. doi: 10.1002/anie.201502813 (PMC4552995; doi:10.1002/anie.201502813)
Supplement: Supplementary file 1 — miscellaneous_information [file anie0054-9830-sd1.pdf]

Angewandte Chemie
Eine Zeitschrift der Gesellschaft Deutscher Chemiker

Supporting Information

Deciphering the Non-Equivalence of Serine and Threonine *O*-Glycosylation Points: Implications for Molecular Recognition of the Tn Antigen by an anti-MUC1 Antibody**

Nuria Martínez-Sáez, Jorge Castro-López, Jessika Valero-González, David Madariaga, Ismael Compañón, Víctor J. Somovilla, Míriam Salvadó, Juan L. Asensio, Jesús Jiménez-Barbero, Alberto Avenoza, Jesús H. Busto, Gonçalo J. L. Bernardes, Jesús M. Peregrina, Ramón Hurtado-Guerrero,* and Francisco Corzana**

anie_201502813_sm_miscellaneous_information.pdf

Reagents and general procedures. Commercial reagents were used without further purification. Solvents were dried and redistilled prior to use in the usual way. ^{1}H and ^{13}C NMR spectra were measured with a 400 MHz spectrometer with TMS as the internal standard. Multiplicities are quoted as singlet (s), broad singlet (br s), doublet (d), doublet of doublets (dd), triplet (t), or multiplet (m). Spectra were assigned using COSY and HSQC. All NMR chemical shifts (δ) were recorded in ppm and coupling constants (*J*) were reported in Hz. The results of these experiments were processed with MestreNova software. High-resolution electrospray mass (ESI) spectra were recorded on a microTOF spectrometer; accurate mass measurements were achieved by using sodium formate as an external reference.

NMR experiments. NMR experiments were performed on a 400 MHz spectrometer at 298 K. Magnitude-mode ge-2D COSY spectra were acquired with gradients by using the *cosygpqf* pulse program with a pulse width of 90º. Phase-sensitive ge-2D HSQC spectra were acquired by using z-filter and selection before t1 removing the decoupling during acquisition by use of the invigpndph pulse program with CNST2 (*J*HC)=145.

2D ROESY experiments. ROESY experiments were recorded on a 400 MHz spectrometer at 298 K and pH 6.5 in H_2O/D_2O (9:1). The experiments were conducted by using phase-sensitive ge-2D ROESY with WATERGATE for H_2O/D_2O (9:1) spectra. NOEs intensities were normalized with respect to the diagonal peak at zero mixing time. Distances involving NH protons were semi-quantitatively determined by integrating the volume of the corresponding cross-peaks. The number of scans used was 16 and the mixing time was 500 ms.

Solid-phase (glyco)peptide synthesis. All (glyco)peptides were synthesized by a stepwise solid-phase peptide synthesis using the Fmoc strategy on Rink Amide MBHA resin (0.1 mmol). The glycosylated amino acid building blocks (2.0 equiv) were synthesized as described in the literature[S1] and manually coupled using HBTU, while the other Fmoc amino acids (10.0 equiv) were automatically coupled on an Applied Biosystems 433A peptide synthesizer using HBTU. The *O*-acetyl groups of $(AcO)_3$GalNAc moiety were removed in a mixture of NH_2NH_2/MeOH (7:3). The (glyco)peptides were then released from the resin, and all acid sensitive side-chain protecting groups were simultaneously removed using TFA 95%, TIS 2.5%, H_2O 2.5%, followed by precipitation with cold diethyl ether. Finally, all (glyco)peptides were purified by HPLC using a Phenomenex Luna C18(2) column (10 μ, 250 mm × 21.2 mm) and a dual absorbance detector. Glycopeptides **m1*** and **m2*** were prepared as described previously by our group.[S2]

Bio-layer Interferometry (BLI). Binding assays were performed on an Octet Red Instrument (fortéBIO). Ligand immobilization, binding reactions, regeneration and washes were conducted in wells of black polypropylene 96-well microplates. Mucins **m1**, **m1***, **m2** and **m2*** (10 mg/mL) were immobilized on amine-reactive biosensors (AR2G biosensors) in 10 mM NaAc pH 5.5 buffer, using 1-ethyl-3-(3-dimethylaminopropyl)-carbodiimide and N-hydroxysuccinimide for 10 min at 1000 rpm at 25 ºC. All biosensors were subsequently modified by a solution of ethanolamine hydrochloride (1M, pH 8.5), followed by regeneration and wash. Binding analysis were carried out at 25 ºC, 1000 rpm in 10 mM sodium phosphate buffer (pH 7.4) containing 150 mM NaCl, with a 120 s of association followed by a 180 s of dissociation. The surface was thoroughly washed with the running buffer without regeneration solution. Data was analyzed using Data Analysis (fortéBIO), with Savitzky-Golay filtering. Binding was fitted to a 2:1 Heterogeneous ligand model, steady state analysis were performed to obtain the binding kinetics contants (K_D).

Enzyme-linked immunosorbent assay (ELISA). The ELISA plate (*Maleic anhydride 96-well plates*) was coated with 100 µL/well of a solution of MUC-1 derivatives (0-1000 nmol/well) in a phosphate buffer (0.2 M, pH 7.2) and incubated overnight at 25 ºC. The unbound sites were then blocked by adding 200 µL/well of blocking buffer (*Thermo Scientific SuperBlock Blocking Buffer*). After 1 h at 25 ºC, the blocking buffer was removed and the plate wells were washed 3 × 200 µL/well with PBST (Phosphate-buffered saline, 0.1 M sodium phosphate, 0.15 M sodium chloride, pH 7.2, containing 0.05% Tween-20 detergent). Next, wells were incubated with monoclonal MUC1 antibody SM3 (100 µL, diluted 1/150 in PBST buffer) for 2 h. After washing with PBST (3 × 200 µL/well, 2 min/well), the wells were incubated with *anti-mouse IgG, (H+L), horseradish peroxidase conjugated* (100 µL, diluted 1/3000 in PBST buffer) for 1 h at 25 ºC. All wells were again washed first with PBST (5 × 350 µL/well, 2 min/well) and then with 350 µL of water. 3,3′,5,5′-Tetramethylbenzidine (TMB) was added (90 µL/well) and after incubation for 10 min, the reaction was terminated with the addition of 50 mL/well of stop solution (1 M H_2SO_4). Absorbance detection of the wells was immediately performed at 450 nm using an ELISA plate reader. Average absorbance intensities of three replicates were plotted against mucins concentrations.

Purification and crystallization of the scFv-SM3. The DNA sequence encoding the SM3 scFv was synthetically made and codon optimized by GenScript for its expression in *Pichia pastoris* (A 51-bp DNA linker containing a sequence *encoding* a short flexible peptide formed by Ser and Gly residues was introduced in between the DNA encoding for the variable regions V_H and V_L of SM3). The resulting plasmid (pUC57_*SM3*) was then used as a template for amplification of the *SM3 scFv* construct encoding amino acids residues of the variable regions V_H and V_L. This construct was amplified using the forward primer,

5'-CGGAATTC*CTCGAG*AAGAGAGAAGCAGAAGCACAGGTCCAACTGCAGGAATCAGGAGG-3',

containing a *Xho*I site (shown in italic letters) and the reverse primer

5'-CGGAATTC*CCGCGG*GTCGAC<u>TTA</u>TCCCAAG-3', containing a recognition sequence for *Sac*II (italic) and a stop codon (underlined). Subsequently the PCR product was digested with *Xho*I and *Sac*II and cloned into pPICZαA (Invitrogen) resulting in the expression plasmid pPICZαA*SM3*. The plasmid was isolated from the *E. coli* strain DH5α, linearised with *Sac*I and used to transform the *Pichia pastoris* strain X33 (Invitrogen) as previously described. Transformants were selected on YPDS plates (1% (w/v) yeast extract, 2% (w/v) peptone, 2% (w/v) dextrose, 1 M sorbitol) supplemented with 100 µg/mL zeocin (InvivoGen). Cells expressing SM3 scFv were grown 24 h at 30 ºC in BMGY medium (1% (w/v) yeast extract, 2% (w/v) peptone, 100 mM potassium phosphate pH 6.0, 1.34% (w/v) yeast nitrogen base and 1% (v/v) glycerol), then centrifuged at 4000 g for 10 minutes. Cells were resuspended in BMM medium (100 mM potassium phosphate pH 6.0, 1.34% (w/v) yeast nitrogen base and 1% (v/v) methanol) and incubated at 18 ºC. Supernatant containing SM3 scFv was collected after 72 h of methanol induction and concentrated to 20–50 mL using a Pellicon XL device (10,000 MWCO, PES membrane; Millipore) then dialyzed against 25 mM Tris HCl pH 8.5.

SM3 ScFv sample was loaded into a HiTrap QFF (GE Healthcare) that had been previously equilibrated with 10 column volumes of 25 mM Tris HCl pH 8.5. The protein was eluted in the presence of a NaCl gradient (from 0 to 1 M) in the above buffer. The fractions containing the protein were then pooled and concentrated to 2.5 mL using centrifugal filter units of 10,000 MWCO (Millipore). Subsequently, gel filtration chromatography was carried out using Superdex 75 XK26/60 column (GE Healthcare, Piscataway, NJ, USA) in 25 mM Tris pH 8.5, 150 mM NaCl. SM3 ScFv was dialysed in 25 mM Tris pH 8.5 and measured by absorbance at 280 nm using an extinction coefficient of 53650 $M^{-1}\cdot cm^{-1}$.

Crystallization. Crystals were grown by sitting drop diffusion at 18 ºC. The drops were prepared by mixing 0.5 µl of protein solution containing 15 mg/mL scFv-SM3 and 10 mM of the different peptides with 0.5 µl of the mother liquor. Crystals of scFv-SM3 with glycopeptides **1*** and **2*** were grown in 20% PEG 5000 monomethyl ether, 0.2 M potasium citrate, 0.1 M MES pH 6.3, and 20% PEG 3350, 0.2 M disodium hydrogen phosphate, respectively. Crystals of scFv-SM3 with the peptide **1** were grown in 20% PEG 3350, 0.2 M diammonium hydrogen citrate pH 5. Finally, crystals with glycopeptide **3*** were grown in 20% PEG 20000, 0.1M bicine pH 9 and 2% 1,4-dioxane. The crystals used in this study were cryoprotected in mother liquor solutions containing 20% ethylenglycol and frozen in a nitrogen gas stream cooled to 100 K.

Structure determination and refinement. Diffraction data of the binary complex was collected in Diamond (Oxford) at beamlines I04 (experiment number MX8035-26) and I02 (experiment number MX10121-2), respectively. All data were processed and scaled using the XDS package[S3] and CCP4[S4] software, relevant statistics are given in Supplementary Table S1. The crystal structures were solved by molecular replacement with Phaser[S4] and using the PDB entry 1SM3 as the template. Initial phases were further improved by cycles of manual model building in Coot[S5] and refinement with REFMAC5.[S6] The final models were validated with PROCHECK,[S7] model statistics are given in Supplementary Table S1. For clarification purposes, the numbering of the amino acids is the same as described for the SM3 Fab. Coordinates and structure factors have been deposited in the Worldwide Protein Data Bank (wwPDB, and see Table S1 for the pdb codes).

Unrestrained Molecular Dynamics (MD) simulations on the scFv-SM3 complexes. The starting geometries for svFc-SM3:**1*** and svFc-SM3:**2*** complexes were generated from the X-ray crystal structures resolved in this work and modified accordingly. Each complex was immersed in a truncated octahedral box with a 10 Å buffer of TIP3P[S8] water molecules. All subsequent simulations were performed using AMBER 12 package[S9] and the *ff14SB* force field, which is an evolution of the Stony Brook modification of the Amber 99 force field force field (*ff99SB*).[S10] This force field was implemented with GLYCAM 06 parameters[S11] to accurately simulate the corresponding glycopeptides. A two-stage geometry optimization approach was performed. The first stage minimizes only the positions of solvent molecules and ions, and the second stage is an unrestrained minimization of all the atoms in the simulation cell. The systems were then heated by incrementing the temperature from 0 to 300 K under a constant pressure of 1 atm and periodic boundary conditions. Harmonic restraints of 30 kcal·mol^{-1} were applied to the solute, and the

Andersen temperature coupling scheme[S12] was used to control and equalize the temperature. The time step was kept at 1 fs during the heating stages. Water molecules are treated with the SHAKE algorithm such that the angle between the hydrogen atoms is kept fixed. Long-range electrostatic effects are modeled using the particle-mesh-Ewald method.[S13] An 8 Å cutoff was applied to Lennard-Jones and electrostatic interactions. Each system was equilibrated for 2 ns with a 2 fs timestep at a constant volume and temperature of 300 K. Production trajectories were then run for additional 25 ns under the same simulation conditions.

MD simulations with time-averaged restraints (MD-tar) in explicit water. MD-tar simulations were performed with AMBER 12 (*ff14SB* force field),[S10] which was implemented with GLYCAM 06 parameters.[S11] Distances derived from NOE cross-peaks were included as time-averaged distance restraints. $< r^{-6} >^{-1/6}$ average was used for the distances. Final trajectories were run using an exponential decay constant of 2000 ps and a simulation length of 20 ns in explicit TIP3P water molecules.

Compound 1

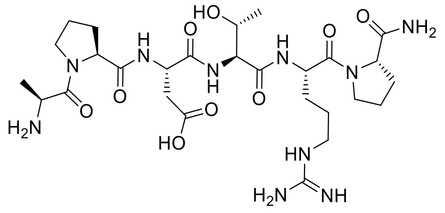

Following SPPS methodology with the adequately protected amino acids, compound **1** was obtained and purified by semi-preparative HPLC. ^{1}H NMR (400 MHz, D_2O) δ (ppm) 1.19 (d, 3H, J = 6.4 Hz, CH_3 $_{Thr4}$), 1.55 (d, 3H, J = 7.0 Hz, CH_3 $_{Ala1}$), 1.62 – 2.13 (m, 10H, 2Hβ $_{Arg5}$, 2Hγ $_{Arg5}$, Hβ $_{Pro2}$, Hβ $_{Pro6}$, 2Hγ $_{Pro2}$, 2Hγ $_{Pro6}$), 2.27 – 2.39 (m, 2H, Hβ $_{Pro2}$, Hβ $_{Pro6}$), 2.85 – 3.05 (m, 2H, 2Hβ $_{Asp3}$), 3.22 (t, 2H, J = 6.7 Hz, 2Hδ $_{Arg5}$), 3.60 – 3.87 (m, 4H, 2Hδ $_{Pro2}$, 2Hδ $_{Pro6}$), 4.18 – 4.27 (m, 1H, Hβ $_{Thr4}$), 4.33 (d, 1H, J = 4.6 Hz, Hα $_{Thr4}$), 4.34 – 4.42 (m, 2H, Hα $_{Ala1}$, Hα $_{Pro2}$), 4.46 – 4.52 (m, 1H, Hα $_{Pro6}$), 4.64 – 4.70 (m, 1H, Hα $_{Arg5}$), 4.73 – 4.80 (m, 1H, Hα $_{Asp3}$). A second set of signals (in a small percentage) is observed. They correspond to the cis disposition of the amide bond of proline residues.[S14] ^{13}C NMR (100 MHz, D_2O) δ (ppm) 15.0 (CH_3 $_{Ala1}$), 18.9 (CH_3 $_{Thr4}$), 24.0, 24.6, 24.7 (Cβ $_{Arg5}$, Cγ $_{Pro2}$, Cγ $_{Pro6}$), 27.5 (Cγ $_{Arg5}$), 29.4, 29.6 (Cβ $_{Pro2}$, Cβ $_{Pro6}$), 35.1 (Cβ $_{Asp3}$), 40.6 (Cδ $_{Arg5}$), 47.7, 47.9 (Cδ $_{Pro2}$, Cδ $_{Pro6}$), 48.1 (Cα $_{Ala1}$), 50.1 (Cα $_{Asp3}$), 51.2 (Cα $_{Arg5}$), 59.0(Cα $_{Thr4}$), 60.3, 60.5 (Cα $_{Pro2}$, Cα $_{Pro6}$), 67.0 (Cβ $_{Thr4}$), 156.8 (CNH $_{Arg5}$) 169.4, 171.4, 171.4, 172.5, 173.7, 173.9, 176.8 (7 CO). Semi-preparative HPLC: Rt = 15.7 min (Phenomenex Luna C18 (2), 21.20×250mm, Grad: acetonitrile/water+0.1% TFA (2:98) → (15:85), 40 min, λ = 212 nm). HRMS ESI+ (m/z) calcd. for $C_{27}H_{47}N_{10}O_9$ $[M+H]^+$ 655.3527, found 655.3546.

^{1}H NMR 400 MHz in D_2O of **compound 1**

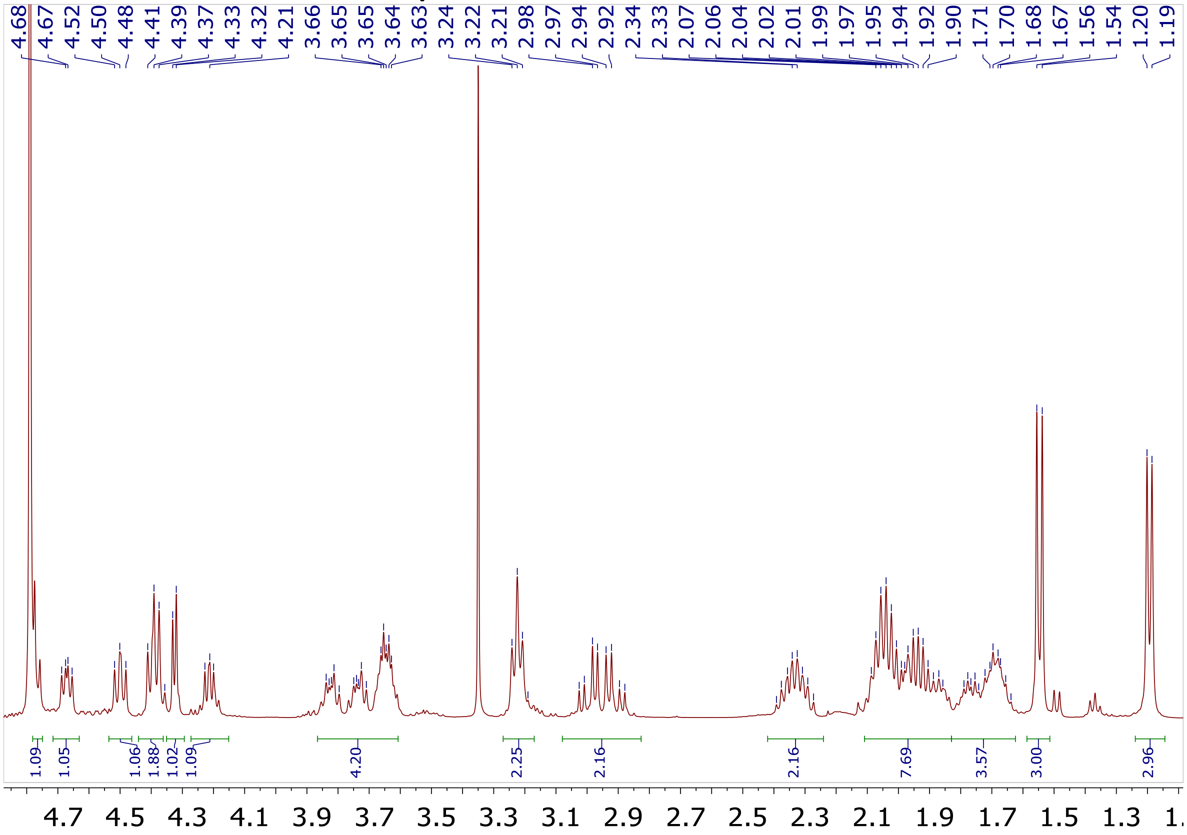

^{13}C NMR 100 MHz in D_2O of **compound 1**

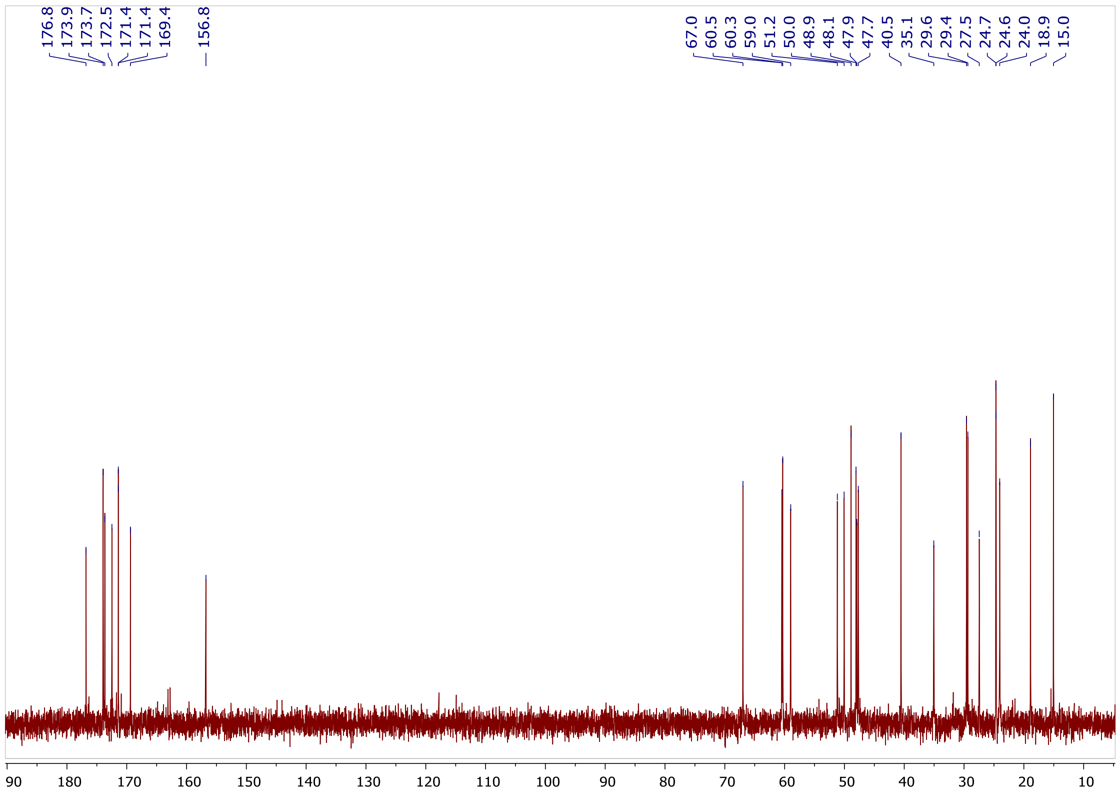

COSY in D_2O of **compound 1**

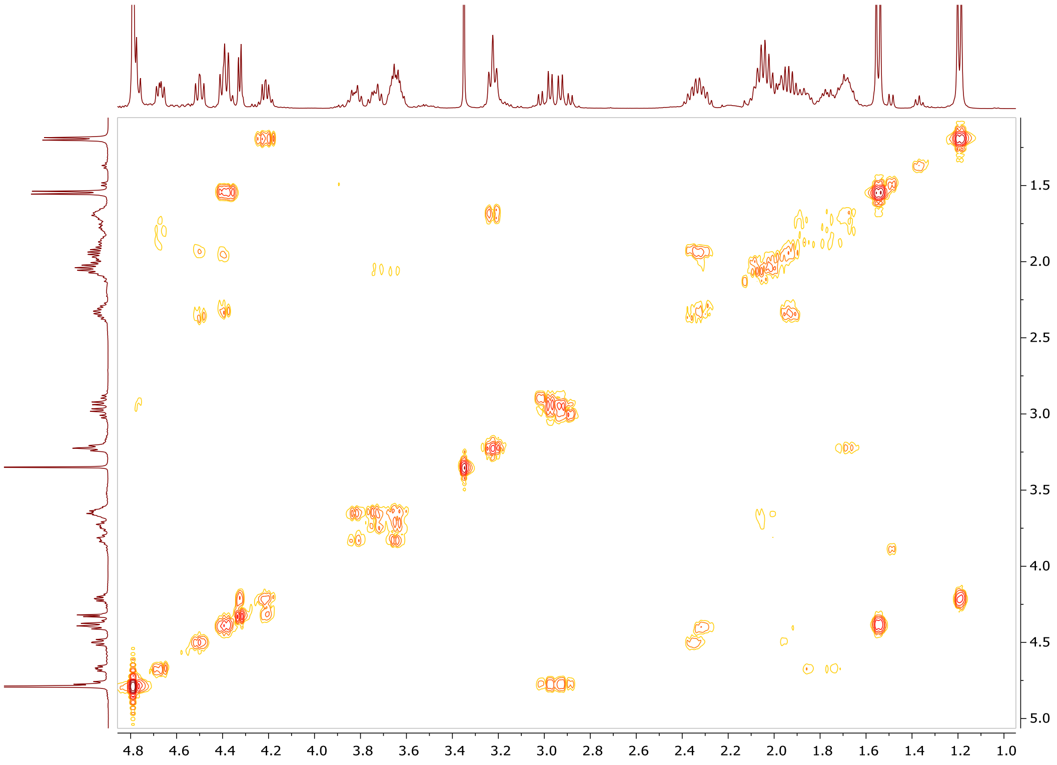

HSQC in D_2O of **compound 1**

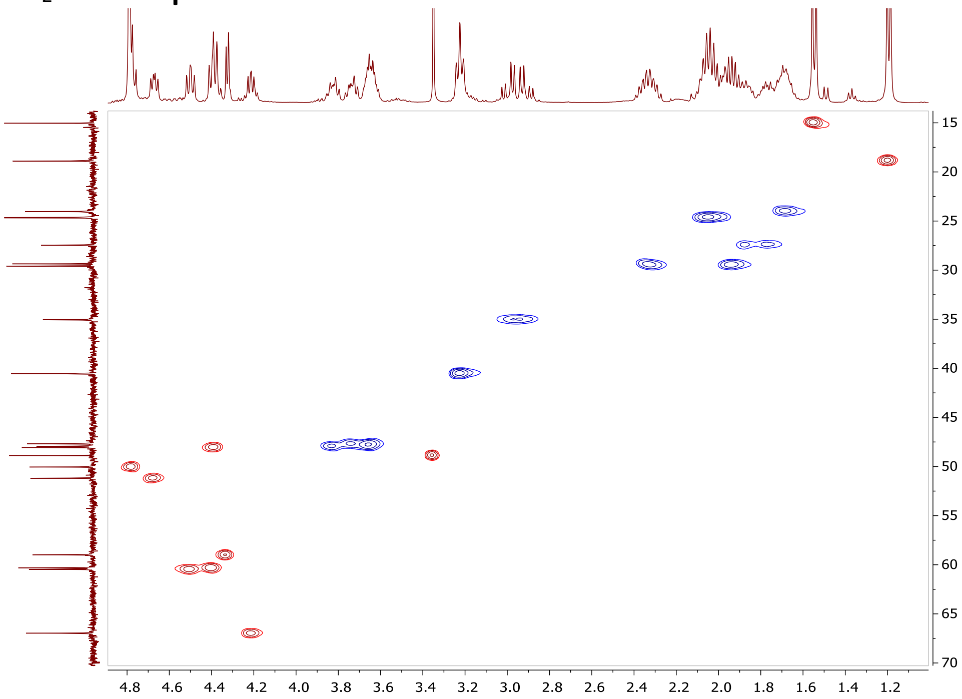

Compound 1*

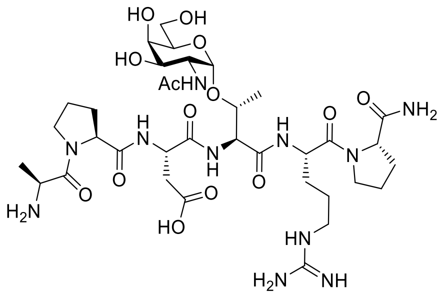

Following SPPS methodology with the adequately protected amino acids, compound **1*** was obtained and purified by semi-preparative HPLC. ^{1}H NMR (400 MHz, D_2O) *δ* (ppm) 1.27 (d, 3H, *J* = 6.3 Hz, CH_3 $_{Thr4}$), 1.54 (d, 3H, *J* = 7.0 Hz, CH_3 $_{Ala1}$), 1.61 – 1.79 (m, 3H, Hβ $_{Arg5}$, 2Hγ $_{Arg5}$), 1.82 – 1.97 (m, 3H, Hβ $_{Pro2}$, Hβ $_{Pro6}$, Hβ $_{Arg5}$), 1.99 – 2.15 (m, 7H, NHCOC$\underline{H}_3$, 2Hγ $_{Pro2}$, 2Hγ $_{Pro6}$), 2.27 – 2.41 (m, 2H, Hβ $_{Pro2}$, Hβ $_{Pro6}$), 2.85 – 3.06 (m, 2H, 2Hβ $_{Asp3}$), 3.24 (t, 2H, *J* = 6.8 Hz, 2Hδ $_{Arg5}$), 3.60 – 3.68 (m, 2H, Hδ $_{Pro2}$, Hδ $_{Pro6}$), 3.68 – 3.78 (m, 4H, Hδ $_{Pro2}$, Hδ $_{Pro6}$, 2H6), 3.84 – 3.89 (dd, 1H, *J* =11.0, 3.1 Hz, H3), 3.97 (d, 1H, *J* = 2.9 Hz, H4), 4.02 (t, 1H, *J* = 6.4 Hz, H5), 4.13 (dd, 1H, *J* = 11.0, 3.7 Hz, H2), 4.26 – 4.43 (m, 3H, Hα $_{Ala1}$, Hβ $_{Thr4}$, Hα $_{Arg5}$), 4.48 – 4.58 (m, 3H, Hα $_{Thr4}$, Hα $_{Pro2}$, Hα $_{Pro6}$), 4.58 – 4.68 (m, 1H, NH $_{Arg5}$), 4.85 – 4.92 (m, 2H, Hα $_{Asp3}$, H1). ^{1}H NMR (400 MHz, H_2O/D_2O, 9:1) δ (ppm) amide protons: 8.61 (t, J = 7.1 Hz, 1H, NH_{Asp3}), 8.35 (d, *J* = 8.9 Hz, 1H, NH_{Thr4}), 8.31 (d, *J* = 7.4 Hz, 1H, NH_{Arg5}), 7.59 (d, *J* = 9.7 Hz, 1H, NH_{GalNAc}). A second set of signals (in a small percentage) is observed. They correspond to the cis disposition of the amide bond of proline residues.[S14] ^{13}C NMR (100 MHz, D_2O) *δ* (ppm) 15.1 (CH_3 $_{Ala1}$), 18.3 (CH_3 $_{Thr4}$), 22.3 (NHCO$\underline{C}H_3$), 24.2, 24.7(Cγ $_{Arg5}$, Cγ $_{Pro2}$, Cγ $_{Pro6}$), 27.4 (Cβ $_{Arg5}$), 29.5, 29.5 (Cβ $_{Pro2}$, Cβ $_{Pro6}$), 35.2 (Cβ $_{Asp2}$), 40.5 (Cδ $_{Arg5}$), 47.7, 47.7 (Cδ $_{Pro}$), 48.0 (Cα $_{Ala1}$), 49.6, 49.9 (C2, Cα $_{Asp3}$), 57.1 (Cα $_{Thr4}$), 60.1 (Cα $_{Pro}$), 60.2 (Cα $_{Arg5}$), 61.3 (C6), 68.2, 68.5, 71.4 (C3, C4, C5), 75.5 (Cβ $_{Thr4}$), 98.6 (C1), 156.8 ($Cζ_{Arg5}$) 169.2, 171.0, 171.1, 172.7, 173.5, 173.8, 174.0, 176.8 (8 CO). Semi-preparative HPLC: Rt = 17.80 min (Phenomenex Luna C18 (2), 21.20×250mm, Grad: acetonitrile/water+0.1% TFA (2:98) → (15:85), 40 min, λ = 212 nm). HRMS ESI+ (m/z) calcd. for $C_{35}H_{60}N_{11}O_{14}$ $[M+H]^+$ 858.4321, found 858.4283.

^1H NMR 400 MHz in D_2O of **compound 1***

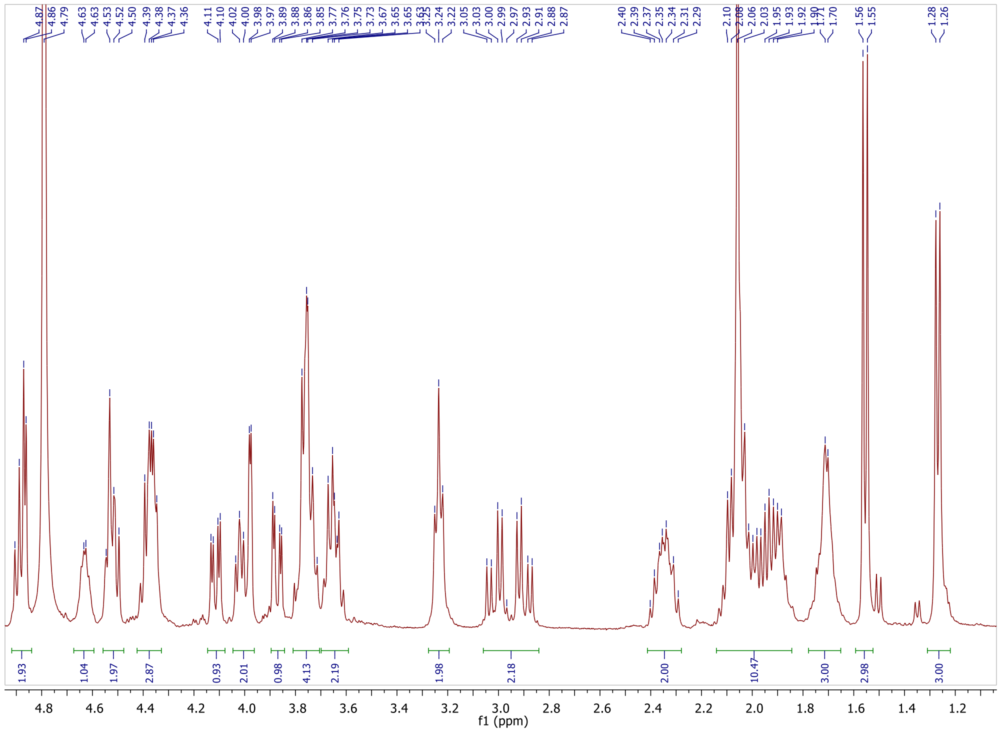

^{13}C NMR 100 MHz in D_2O of **compound 1***

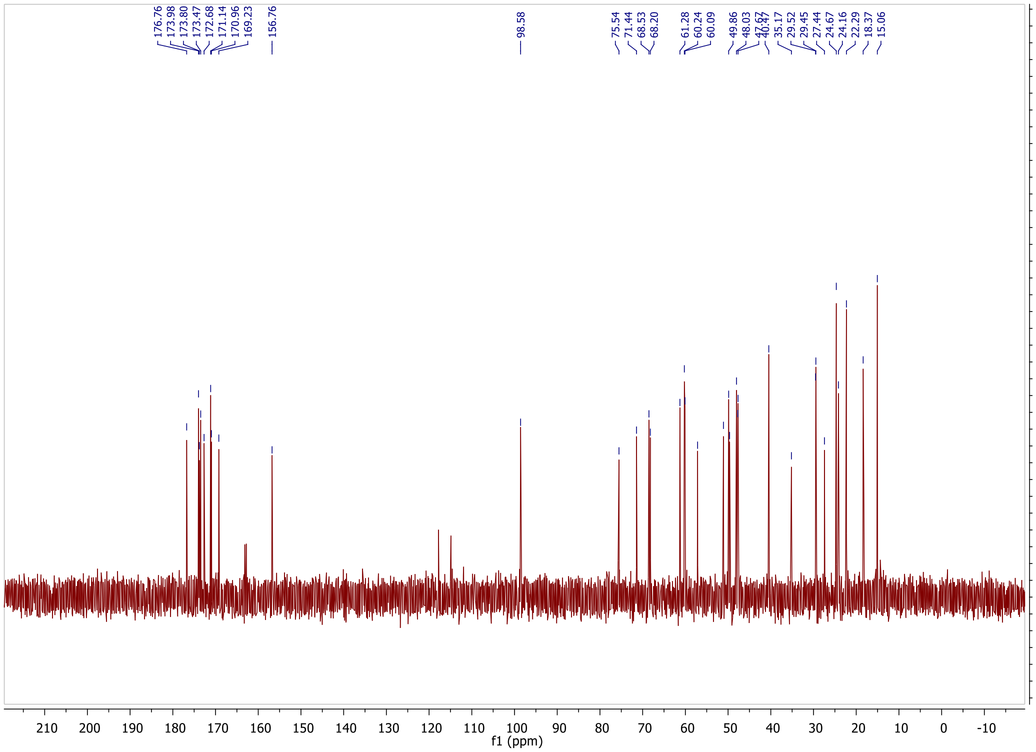

COSY in D_2O of **compound 1***

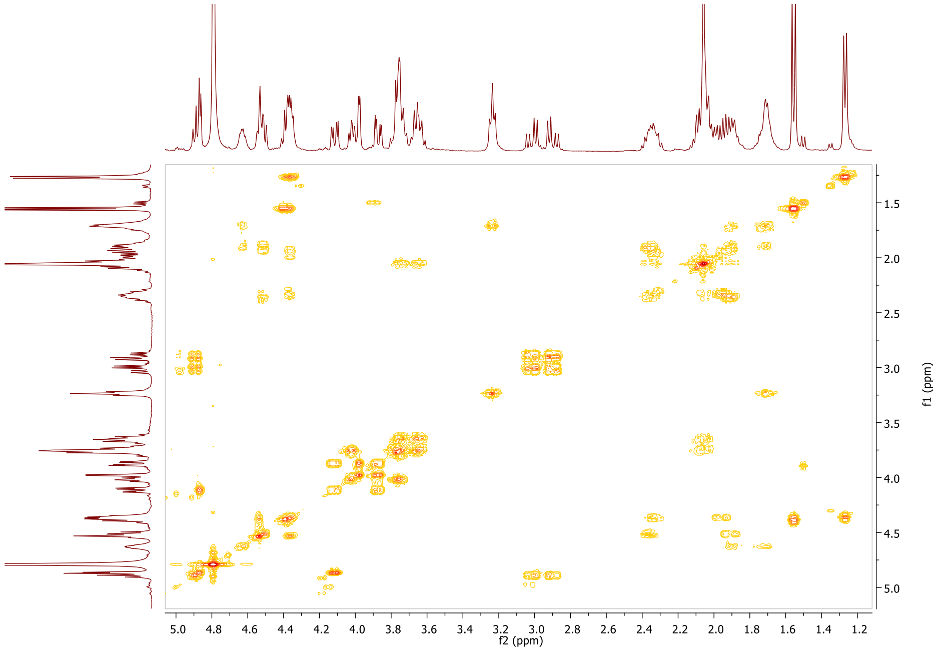

HSQC in D_2O of **compound 1***

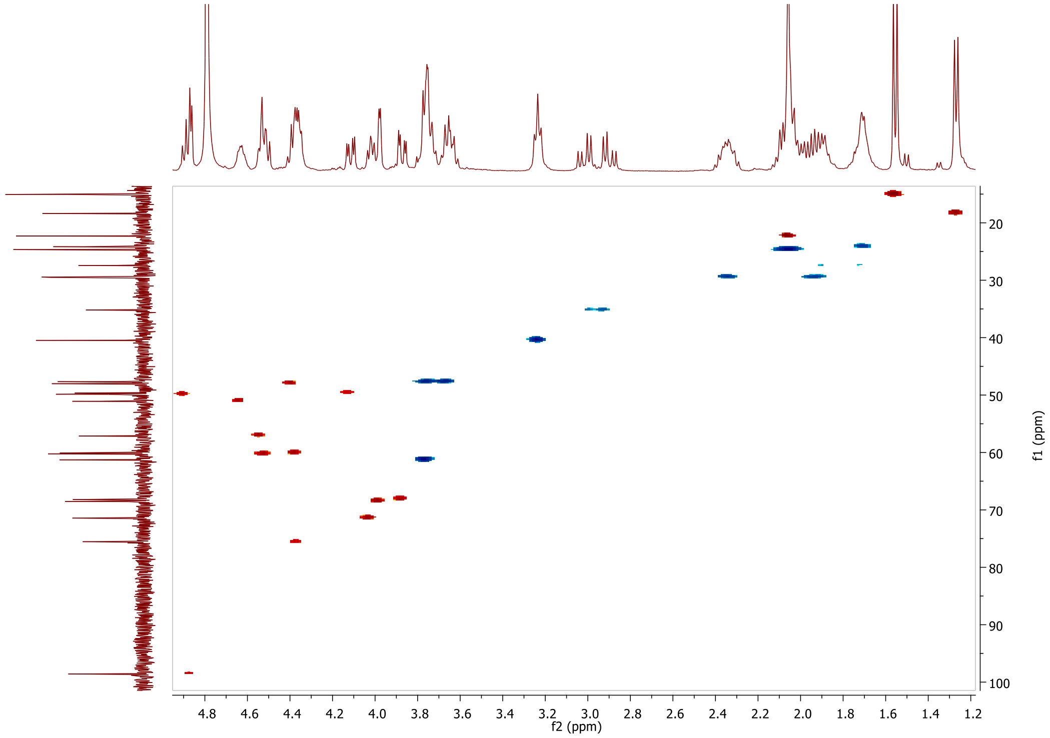

Compound 2*

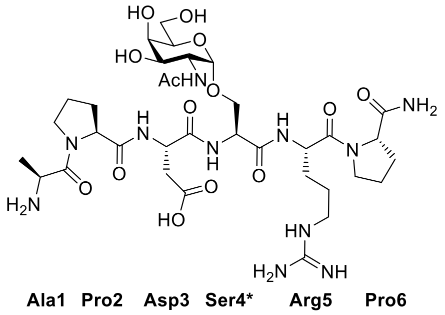

Following SPPS methodology with the adequately protected amino acids, compound **2*** was obtained and purified by semi-preparative HPLC. ^{1}H NMR (400 MHz, D_2O) *δ* (ppm) 1.54 (d, 3H, *J* = 7.1 Hz, CH_3 $_{Ala1}$), 1.61 – 1.82 (m, 3H, Hβ $_{Arg5}$, 2Hγ $_{Arg5}$), 1.83 – 2.14 (m, 10H, Hβ $_{Pro2}$, Hβ $_{Pro6}$, Hβ $_{Arg5}$, NHCOC$\underline{H}_3$, 2Hγ $_{Pro2}$, 2Hγ $_{Pro6}$), 2.26 – 2.40 (m, 2H, Hβ $_{Pro2}$, Hβ $_{Pro6}$), 2.83 – 3.03 (m, 2H, 2Hβ $_{Asp3}$), 3.18-3.28 (m, 2H, 2Hδ $_{Arg5}$), 3.58 – 4.00 (m, 11H, 2Hδ $_{Pro2}$, 2Hδ $_{Pro6}$, 2H6, H3, H4, H5, 2Hβ $_{Ser4}$), 4.18 (dd, 1H, *J* = 11.0, 3.7 Hz, H2), 4.34 – 4.42 (m, 2H, Hα $_{Ala1}$, Hα $_{Pro}$), 4.50 (dd, 1H, *J* = 8.3, 6.3 Hz, Hα $_{Pro}$), 4.58 – 4.65 (m, 1H, Hα $_{Ser4}$) 4.66 – 4.75 (m, 2H, Hα $_{Arg5}$, Hα $_{Asp3}$), 4.88 (d, 1H, *J* = 3.8 Hz, H1). ^{1}H NMR (400 MHz, H_2O/D_2O, 9:1) δ (ppm) amide protons: 8.65 (t, J = 6.7 Hz, 1H, NH_{Asp3}), 8.49 (d, *J* = 7.3 Hz, 1H, NH_{Arg5}), 8.41 (d, *J* = 7.2 Hz, 1H, NH_{Ser4}), 7.94 (d, *J* = 9.4 Hz, 1H, NH_{GalNAc}). A second set of signals (in a small percentage) is observed. They correspond to the cis disposition of the amide bond of proline residues.[S14] ^{13}C NMR (125 MHz, D_2O) *δ* (ppm) 15.0 (CH_3 $_{Ala1}$), 22.0 (NHCO$\underline{C}H_3$), 24.0, 24.7(Cγ $_{Arg5}$, Cγ $_{Pro2}$, Cγ $_{Pro6}$), 27.5 (Cβ $_{Arg5}$), 29.3, 29.5 (Cβ $_{Pro2}$, Cβ $_{Pro6}$), 35.4 (Cβ $_{Asp2}$), 40.4 (Cδ $_{Arg5}$), 47.7, 47.8 (Cδ $_{Pro}$), 48.0 (Cα $_{Ala1}$), 49.6, (C2), 50.2 (Cα $_{Asp3}$), 51.1 (Cα $_{Arg5}$) 53.2 (Cα $_{Ser4}$), 60.2, 60.2 (2Cα $_{Pro}$), 61.0 (C6), 66.7 (Cβ $_{Ser4}$), 67.7, 68.3, 71.3 (C3, C4, C5), 97.7 (C1), 156.7, 169.2, 170.8, 171.2, 172.3, 173.6, 174.4, 176.7 (CO). HPLC Semi-preparative HPLC: Rt = 17.30 min (Phenomenex Luna C18 (2), 21.20×250mm, Grad: acetonitrile/water+0.1% TFA (2:98) → (15:85), 40 min, λ = 212 nm). HRMS ESI+ (m/z) calcd. for $C_{34}H_{58}N_{11}O_{14}$ $[M+H]^+$ 844.4159, found 844.4182.

^{1}H NMR 400 MHz in D_2O of **compound 2***

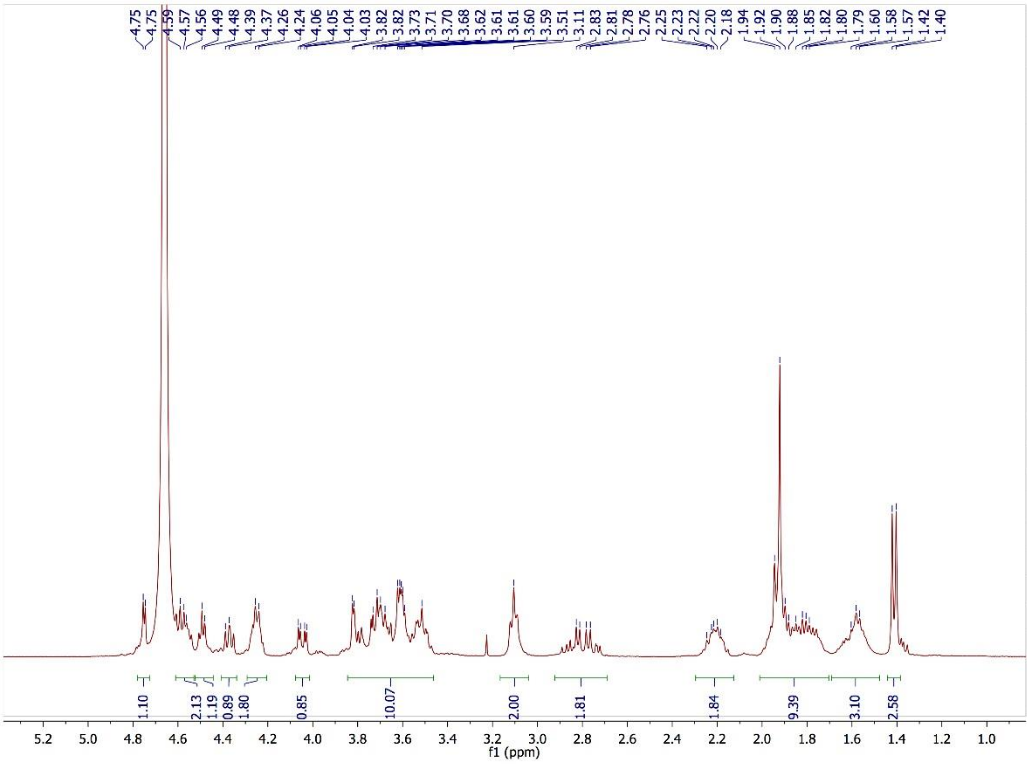

COSY in D_2O of **compound 2***

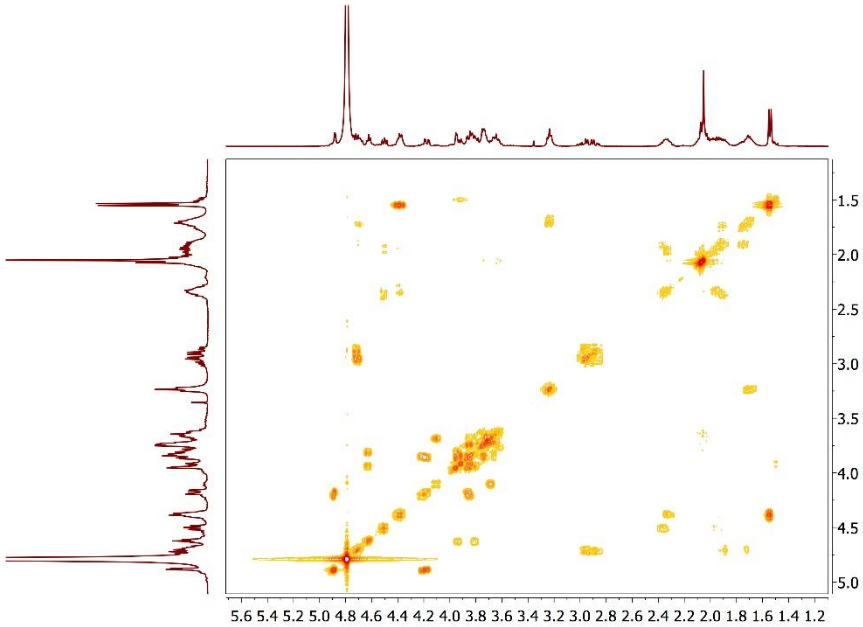

^{13}C NMR 125 MHz in D_2O of **compound 2***

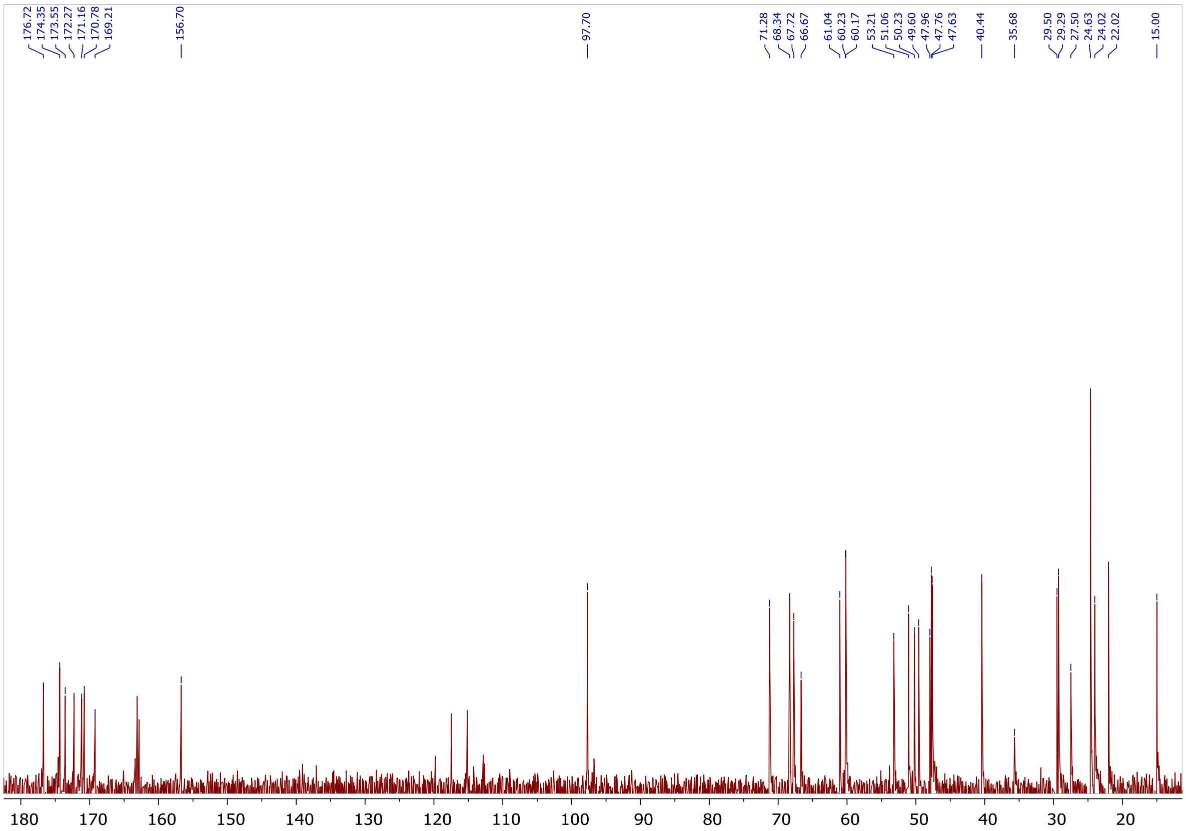

HSQC in D_2O of **compound 2***

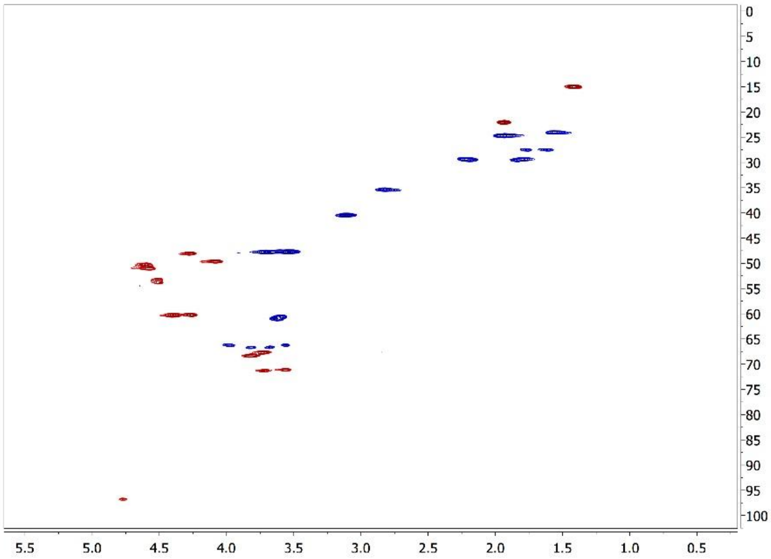

Compound 3*

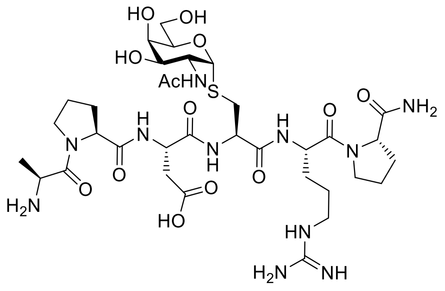

Following SPPS methodology with the adequately protected amino acids, compound **3*** was obtained and purified by semi-preparative HPLC. ^{1}H NMR (400 MHz, D_2O) δ (ppm) 1.55 (d, J = 7.0 Hz, 3H, 3Hβ_{Ala1}), 1.82–1.60 (m, 4H, Hγ_{Pro2}, Hγ_{Pro6}, 2Hγ_{Arg5}), 2.14–1.91 (m, 9H, NHCO$\underline{CH_3}$, 2Hβ_{Arg5}, Hβ_{Pro2}, Hβ_{Pro6}, Hγ_{Pro2}, Hγ_{Pro6}), 2.45–2.25 (m, 2H, Hβ_{Pro2}, Hβ_{Pro6}), 3.16–2.83 (m, 4H, 2Hβ_{Asp3}, 2Hβ_{Cys4}), 3.30–3.18 (m, 2H, 2Hδ_{Arg5}), 3.85–3.59 (m, 7H, 2Hδ_{Pro2}, 2Hδ_{Pro6}, 2H6, H3), 4.27–4.17 (m, 1H, H5), 4.00 (d, J = 2.9 Hz, 1H, H4), 4.44–4.35 (m, 3H, H2, Hα_{Pro}, Hα_{Ala1}), 4.54–4.46 (m, 1H, Hα_{Pro}), 4.65–4.57 (m, 1H, Hα_{Cys4}), 4.68 (d, J = 3.9 Hz, 1H, Hα_{Arg5}), 5.58 (d, J = 5.4 Hz, 1H, H1), 4.73 (t, J = 6.7 Hz, 1H, Hα_{Asp3}). ^{1}H NMR (400 MHz, H_2O/D_2O, 9:1) δ (ppm) amide protons: 8.67 (t, J = 6.9 Hz, 1H, NH$_{Asp3}$), 8.40 (d, J = 7.5 Hz, 1H, NH$_{Cys4}$), 8.36 (d, J = 7.4 Hz, 1H, NH$_{Arg5}$), 8.20 (d, J = 8.2 Hz, 1H, NH$_{GalNAc}$). A second set of signals (in a small percentage) is observed. They correspond to the cis disposition of the amide bond of proline residues.[S14] ^{13}C NMR (100 MHz, D_2O) δ (ppm) 15.1 (Cβ_{Ala1}), 21.9 (NHCO$\underline{C}$H$_3$), 24.1 (Cγ_{Arg5}), 24.6 (Cβ_{Arg5}), 24.7(Cγ_{Pro}), 27.7 (Cγ_{Pro}), 29.3 (Cβ_{Pro}), 29.6 (Cβ_{Pro}), 32.6 (Cβ_{Cys4}), 35.2 (Cβ_{Asp3}), 40.6 (Cδ_{Arg5}), 47.7 (Cδ_{Pro}), 47.9 (Cδ_{Pro}), 48.0 (Cα_{Ala1}), 50.0 (C2), 50.1 (Cα_{Asp3}), 51.1 (Cα_{Arg5}), 53.7 (Cα_{Cys4}), 60.3 (Cα_{Pro} , Cα_{Pro}), 61.1 (C6), 67.6 (C3),68.4 (C4), 71.9 (C5), 85.4 (C1), 156.8 (Cζ_{Arg5}), 169.2 171.1, 171.4, 171.9, 173.6, 173.9, 174.6, 176.8 (8 CO). HPLC Semi-preparative HPLC: Rt = 20.70 min (Phenomenex Luna C18 (2), 21.20×250mm, Grad: acetonitrile/water+0.1% TFA (2:98) → (15:85), 40 min, λ = 212 nm). HRMS ESI+ (m/z) calcd. for $C_{34}H_{58}N_{11}O_{13}S$ $[M+H]^+$ 860.3938, found 860.3936.

^{1}H NMR 400 MHz in D_2O of **compound 3***

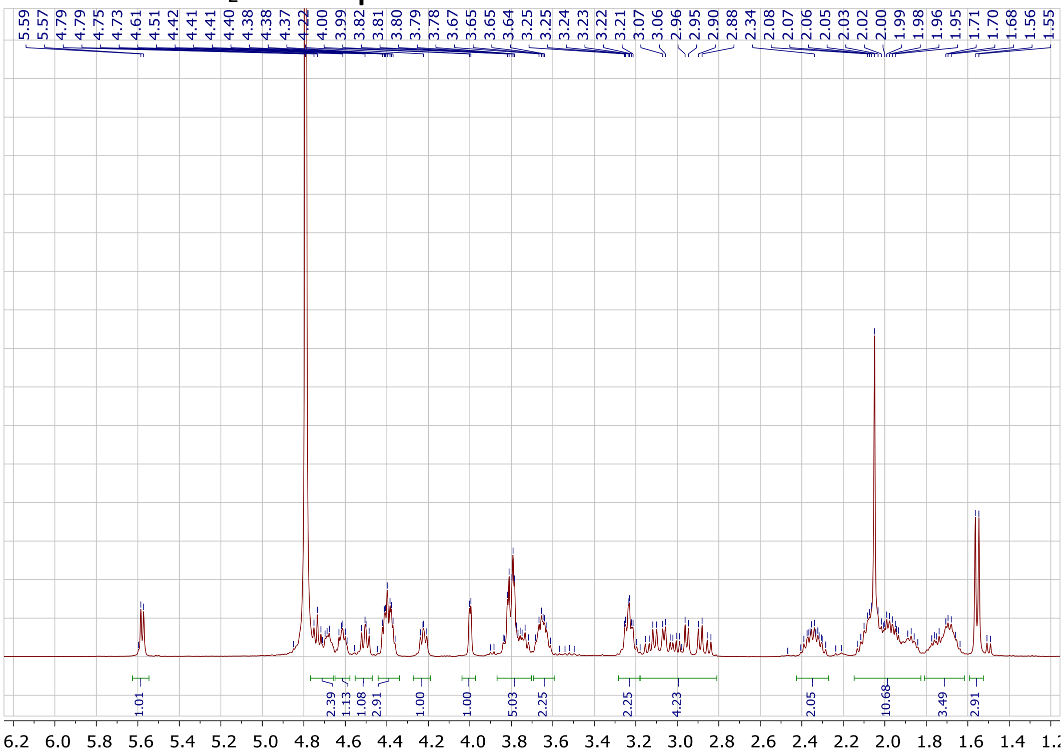

^{13}C NMR 100 MHz in D_2O of **compound 3***

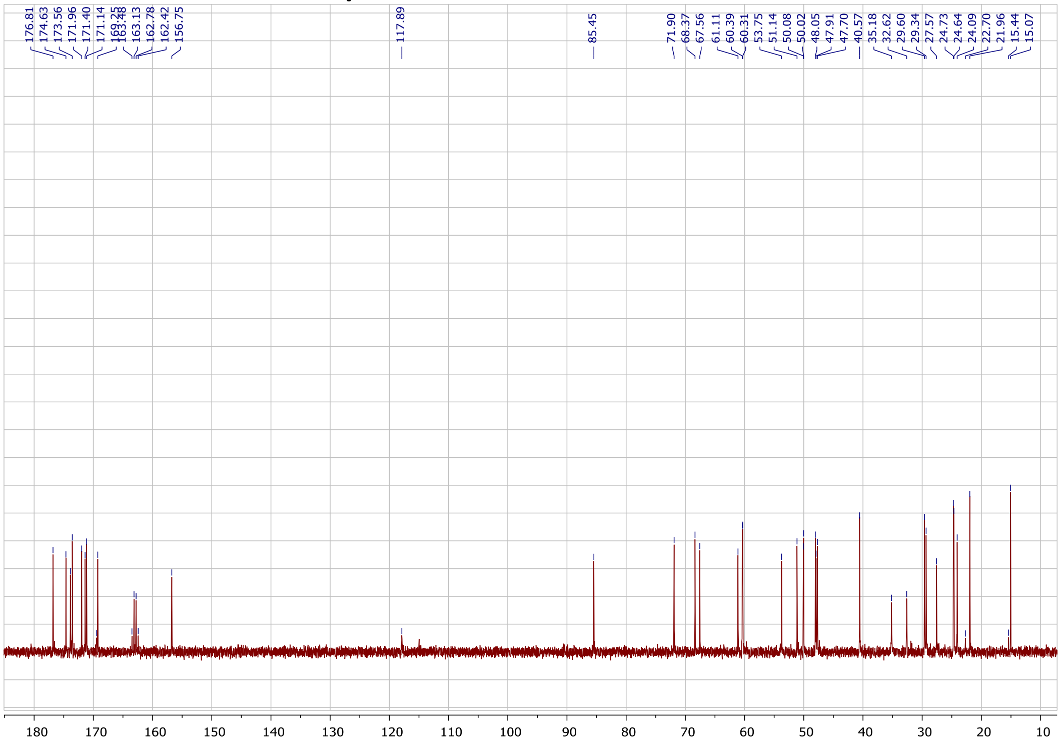

COSY in D_2O of **compound 3**[*]

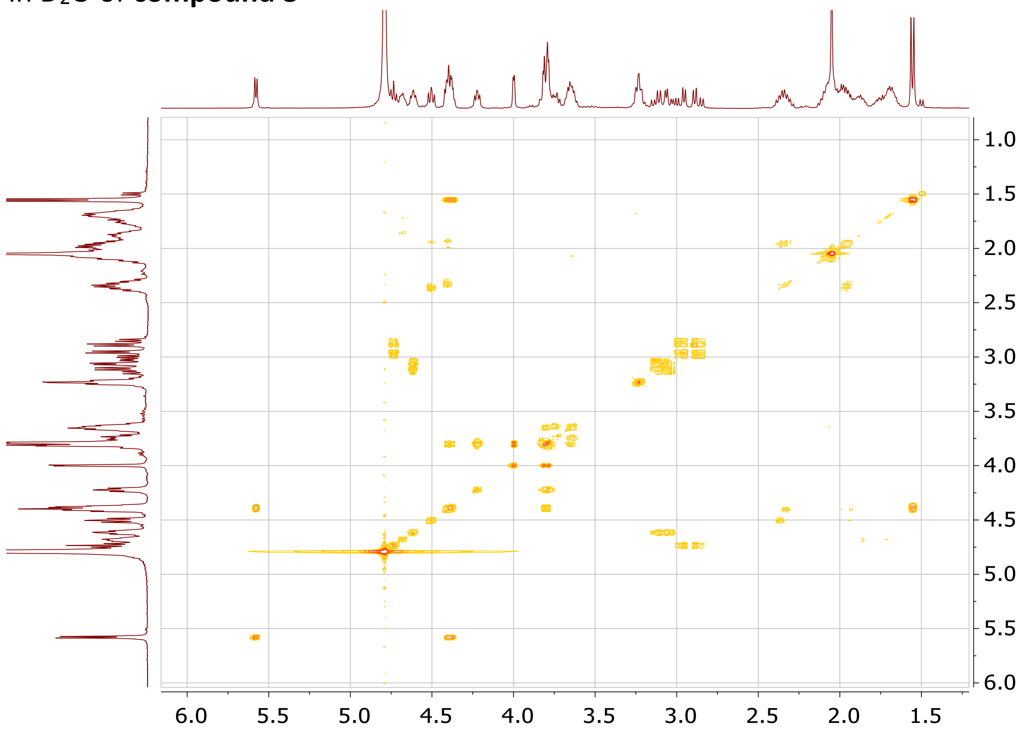

HSQC in D_2O of **compound 3**[*]

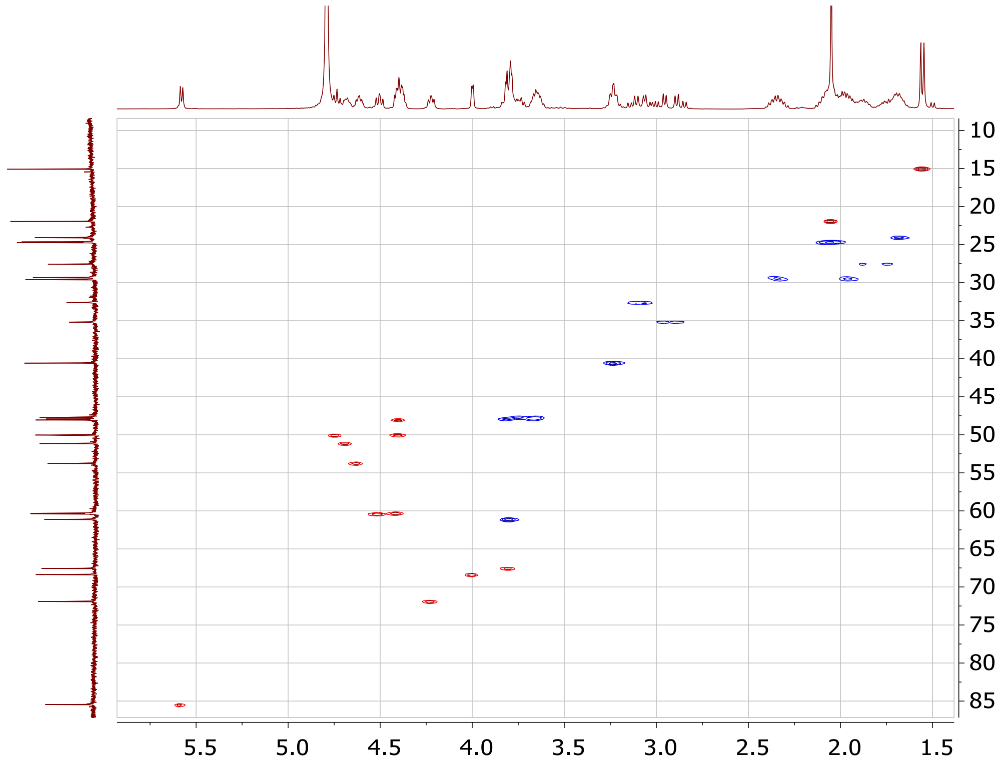

Compound **m1:**

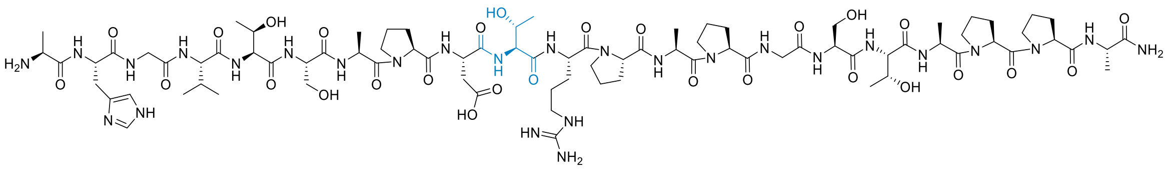

HRMS (ESI+) m/z: calcd. for $C_{83}H_{135}N_{27}O_{28}$: $[M+2H]^{2+}$: 978.9906 found: 978.9917.

Semi-preparative HPLC: R_t = 19.15 min (Phenomenex Luna C18 (2), 21.20×250mm, Grad: acetonitrile/water+0.1% TFA (5:95) → (23:77), 20 min, λ = 212 nm).

Compound **m2:**

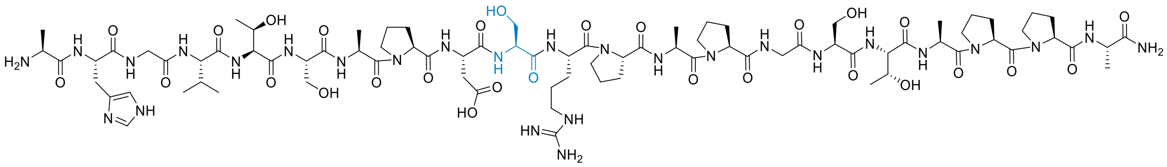

HRMS (ESI+) m/z: calcd. for $C_{82}H_{133}N_{27}O_{28}$: $[M+2H]^{2+}$: 971.9828 found: 971.9837.

Semi-preparative HPLC:

R_t = 18.93 min (Phenomenex Luna C18 (2), 21.20×250mm, Grad: acetonitrile/water+0.1% TFA (5:95) → (23:77), 20 min, λ = 212 nm).

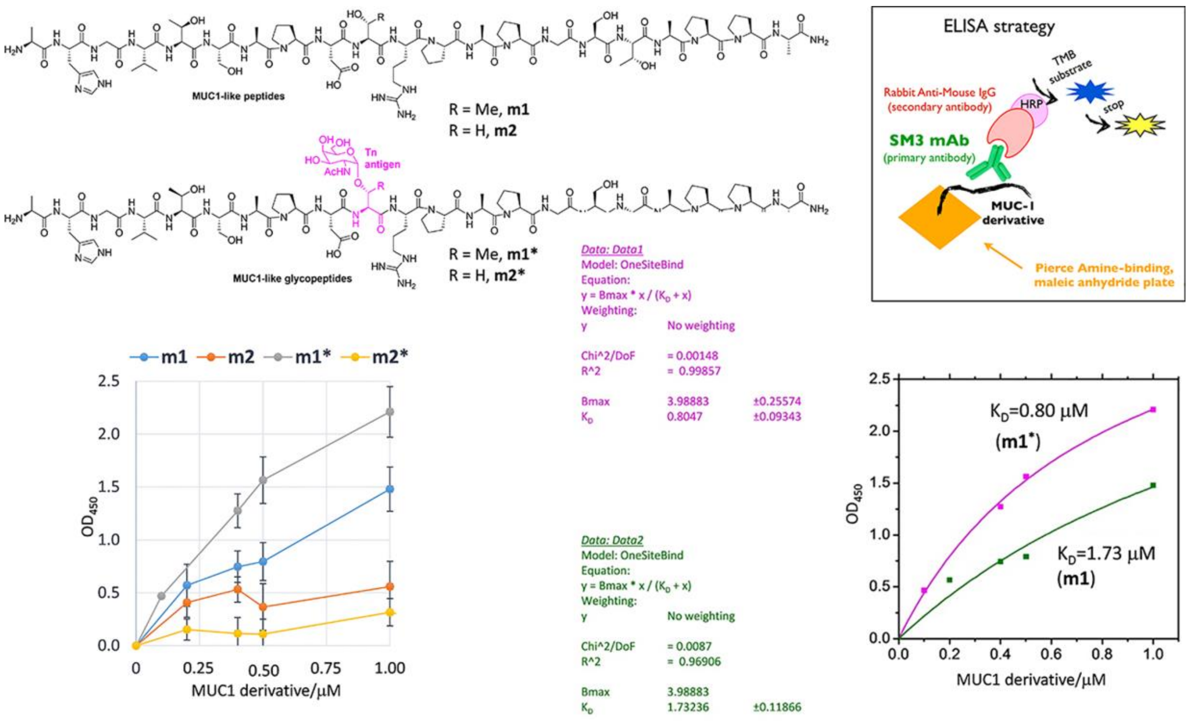

Figure S1. Binding curves for mucin derivatives with the commercial SM3 antibody using a one-site binding mode, together with the schematic representation of the procedure used in ELISA tests. Absorbance signals are the average of three replicate wells. The K_D constants could be determined for **m1** and **m1*** derivatives. The commercially available SM3 antibody has around 2-fold higher affinity to glycopeptide **m1*** than to the corresponding naked peptide **m1**. This result is in agreement with the previously reported data.[S15]

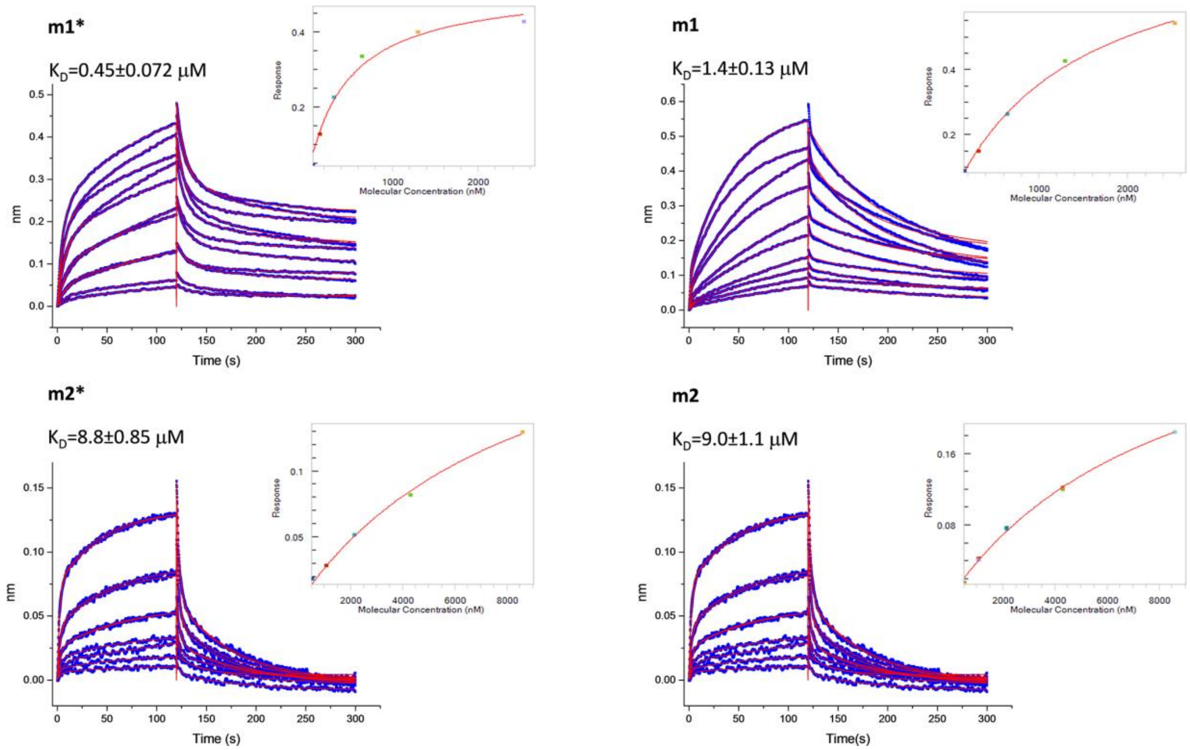

Figure S2. Bio-Layer Interferometry (BLI) curves (in blue) and fitting curves (in red) obtained for **m1**, **m2**, **m1*** and **m2*** with scFv-SM3, together with the K_D constants derived from BLI experiments.

Table S1. Data collection and refinement statistics. Values in parentheses refer to the highest resolution shell. Ramachandran plot statistics were determined with PROCHECK.

	scFv-1SM3 in complex with **1**	scFv-1SM3 in complex with **1***	scFv-1SM3 in complex with **2***	scFv-1SM3 in complex with **3***
Space group	$P2_1$	$P2_12_12_1$	$P2_12_12_1$	$P2_1$
Wavelength (Å)	0.97	0.97	0.97	0.97
Number of molecules in the asymmetric unit (AU)	1	1	1	1
Resolution (Å)	20-1.65 (1.74–1.65)	45-1.70 (1.79–1.70)	20-1.88 (1.98–1.88)	64.59-1.79 (1.88–1.79)
Cell dimensions (Å)	a = 45.85<br>b = 35.49<br>c = 69.52	a = 35.42<br>b = 68.80<br>c = 90.41	a = 33.18<br>b = 66.01<br>c = 90.29	a = 46.08<br>b = 35.19<br>c = 66.29
Unique reflections	26087	25092	16818	19905
Completeness	97.1 (95.8)	100 (99.9)	99.9 (99.9)	99.6 (99.9)
R_{sym}	0.065 (0.279)	0.051 (0.507)	0.083 (0.550)	0.107 (0.597)
$I/\sigma(I)$	13.6 (4.0)	18.9 (3.0)	10.4 (2.4)	7.6 (2.4)
Redundancy	3.8 (3.7)	5.4 (5.5)	6.2 (5.6)	3.6 (3.7)
R_{work} / R_{free}	0.166/0.197	0.175/0.216	0.206/0.261	0.181/0.200
RMSD from ideal geometry, bonds (Å)	0.010	0.011	0.015	0.010
RMSD from ideal geometry, angles (º)	1.493	1.566	1.829	1.520
$\langle B \rangle$ protein (Å^2)	13.97	24.72	35.26	26.06
$\langle B \rangle$ peptide/glycopeptide (Å^2)	13.70	33.56	49.38	35.21
$\langle B \rangle$ solvent (Å^2)	23.49	34.66	38.35	32.77
Ramachandran plot:				
• Most favoured (%)	95.45	95.07	90.91	95.02
• Additionally allowed (%)	3.64	3.14	6.82	3.62
• Disallowed (%)	0.91	1.79	2.27	1.36
PDB ID	5a2j	5a2k	5a2i	5a2l

For clarification purposes, the numbering and labels of the residues in our pdbs are slightly different to the reported one for the PDB entry 1SM3.[S16] In our case, both chains L and H have been merged into one chain (H). Residues from 1000 onwards belong to the chain L in the PDB entry 1SM3.The numbering of compounds **1**,**1***, **2*** and **3*** in chain P is also different to 1SM3 entry.

Table S2. Hydrogen bonds found in the X-ray structures between compound **1** and scFv-SM3.

peptide	scFv-SM3	Hydrogen bond	distances (Å)
**Ala1**	Ser^{93L}	N-water-O	2.77 ,2.75
	Arg^{52H}	O-water-NH1	3.75 , 3.03
	Arg^{52H}	O-water-NH2	3.75 , 3.40
	Trp^{91L}	O-water-Nε	2.77 , 2.88
**Asp3**	Gln^{97H}	O-Nε	2.90
	Trp^{33H}	Oδ2-N	2.78
	Gly^{96H}	O-water-N	2.95 , 2.70
	Gly^{96H}	Oδ2-water-N	3.36 , 2.70
	Asn^{31H}	Oδ1-water-O	2.61 , 2.74
	Asn^{31H}	Oδ2-water-O	3.41 , 2.74
	Trp^{33H}	Oδ2-water-N	2.74 , 3.42
	Trp^{33H}	Oδ2-water-O	2.74 , 2.66
	Val^{95H}	Oδ2-water-N	2.74 , 2.92
**Arg5**	Asn^{31H}	Nε-O	3.12
	Gly^{96H}	O-water-N	2.73 , 2.70
**Pro6**	Tyr^{32H}	O-Oη	2.57

Table S3. Hydrogen bonds found in the X-ray structures between compound **1*** and scFv-SM3.

glycopeptide	scFv-SM3	Hydrogen bond	distances (Å)
**Ala1**	Tyr^{32H}	N-Oη	2.81
	Arg^{52H}	O-water-NH2	2.67 , 2.86
**Asp3**	Gln^{97H}	O-Nε	2.94
	Trp^{33H}	Oδ2-N	2.86
	Gly^{96H}	O-water-N	2.87 , 2.86
	Gly^{96H}	O-water-N	3.45 , 2.86
	Asn^{31H}	Oδ1-water-O	2.69 , 2.74
	Asn^{31H}	Oδ2-water-O	3.32 , 2.74
	Trp^{33H}	Oδ2-water-N	2.74 , 3.47
	Trp^{33H}	Oδ2-water-O	2.74 , 2.63
**Arg5**	Asn^{31H}	Nε-O	3.21
	Asn^{31H}	NH1-Nδ	3.33
	Gly^{96H}	O-water-N	2.66 , 2.69
**Pro6**	Tyr^{32H}	O-Oη	2.57
**GalNAc**	Tyr^{32L}	O6- Oη	3.72

Table S4. Hydrogen bonds found in the X-ray structures between compound **2*** and scFv-SM3.

glycopeptide	scFv-SM3	Hydrogen bond	distances (Å)
**Ala1**	Tyr^{32H}	N-Oη	3.33
**Asp3**	Gln^{97H}	O-Nε	2.92
	Trp^{33H}	Oδ2-N	2.77
	Gly^{96H}	O-water-N	2.98 , 2.92
	Trp^{33H}	Oδ2-water-N	2.90 , 3.42
	Trp^{33H}	Oδ2-water-O	2.90 , 2.55
	Gly^{96H}	Oδ1-water-N	3.17 , 2.92
	Gly^{96H}	Oδ2-water-N	3.23 , 2.92
	Asn^{31H}	Oδ1-water-O	2.57 , 2.71
	Asn^{31H}	Oδ2-water-O	3.17 , 2.71
**Arg5**	Asn^{31H}	NH1-Nδ2	2.96
	Tyr^{32H}	NH1-Oη	2.74
	Gly^{96H}	O-water-N	2.73 , 2.92
**Pro6**	Tyr^{32H}	O-Oη	2.85
**GalNAc**	Trp^{33H}	O6-water-Nε	3.28 , 2.67
	Asn^{31H}	O6-water-O	3.14 , 2.71

Table S5. Hydrogen bonds found in the X-ray structures between compound **3*** and scFv-SM3.

glycopeptide	scFv-SM3	Hydrogen bond	distances (Å)
**Ala1**	Tyr^{32H}	N-Oη	3.46
	Arg^{52H}	O-water-NH1	2.64 , 3.34
	Arg^{52H}	O-water-NH2	2.64 , 2.76
**Asp3**	Gln^{97H}	O-Nε	2.94
	Trp^{33H}	Oδ2-N	2.86
	Asn^{31H}	Oδ1-water-O	2.66 , 2.61
	Asn^{31H}	Oδ2-water-O	3.30 , 2.61
	Trp^{33H}	Oδ2-water-N	2.79 , 3.50
	Trp^{33H}	Oδ2-water-O	2.79 , 2.59
	Val^{95H}	Oδ2-water-N	2.79 , 2.91
**Arg5**	Asn^{31H}	NH1-O	2.73
	Asn^{31H}	NH1-Oδ1	3.40
**Pro6**	Tyr^{32H}	O-Oη	2.95
**GalNAc**	Asn^{31H}	O6-water-O	3.23 , 2.61

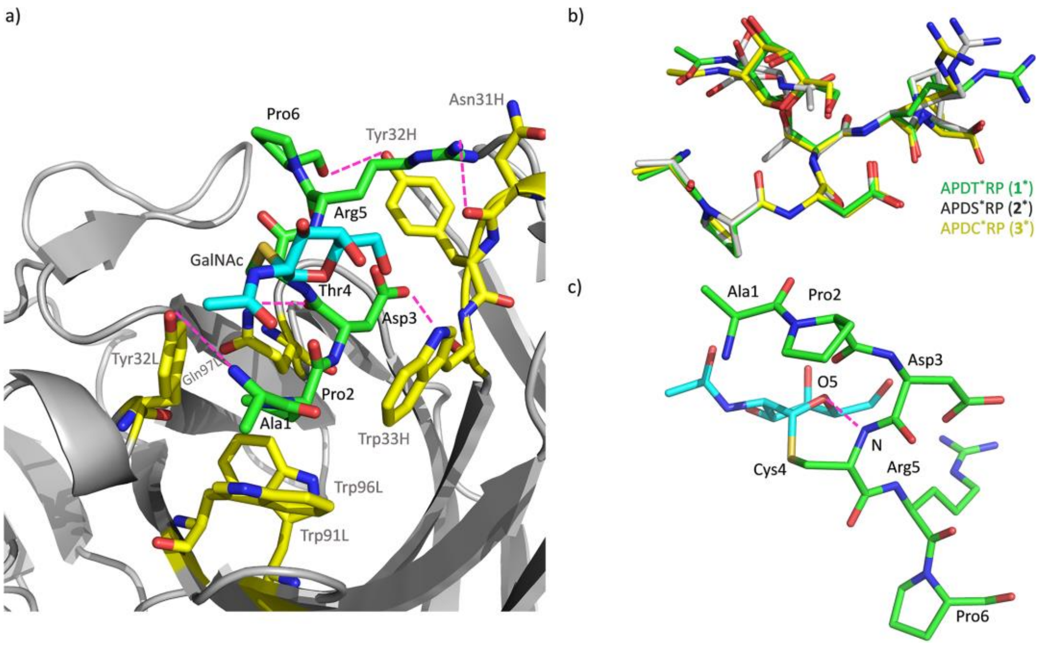

Figure S3. (a) Key binding interactions of glycopeptide **3*** with scFv-SM3 antibody in the X-ray crystal structure. Peptide and GalNAc carbon atoms are in green and cyan, respectively. Carbon atoms of key residues of SM3 are in yellow. Interactions between GalNAc unit and SM3 surface are in pink. (b) Superposition of the peptide backbone of compounds **1***, **2*** and **3*** in complex to scFv-SM3. (c) Conformation of glycopeptide **3*** in complex with scFv-SM3 antibody, together with the geometry of the glycosidic linkage and the hydrogen bond established with Cys residue.

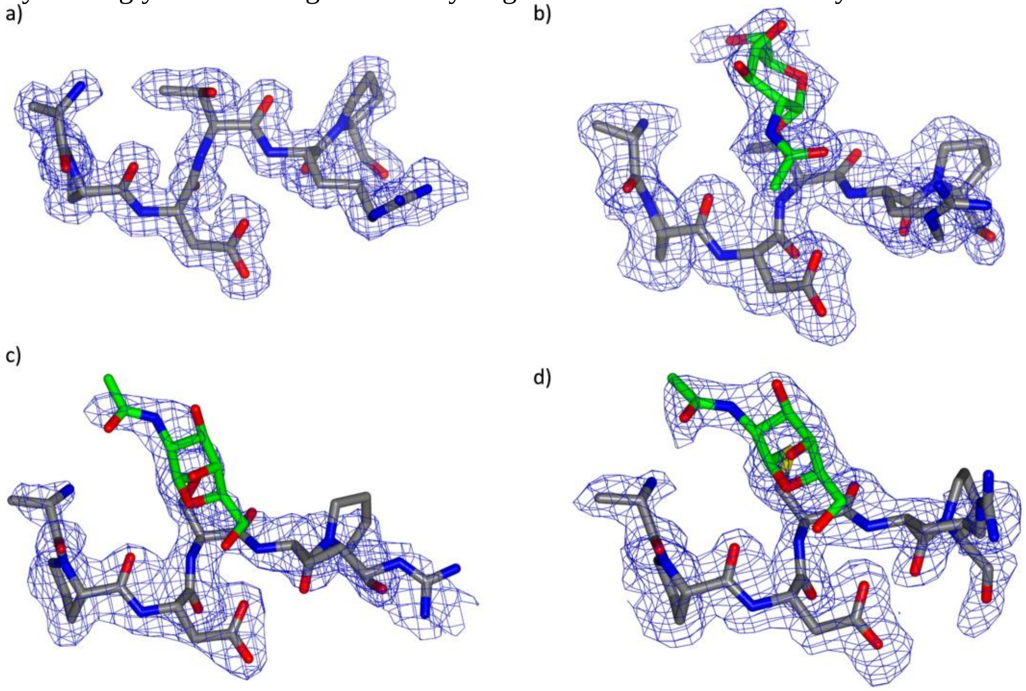

Figure S4. Electron density maps are F_O–F_C syntheses (blue) contoured at 2.0 σ for peptide **1** (a) and glycopeptides **1*** (b) and **2*** (c) and **3*** (d). The amino acid residues and the GalNAc moiety are coloured in grey and green, respectively.

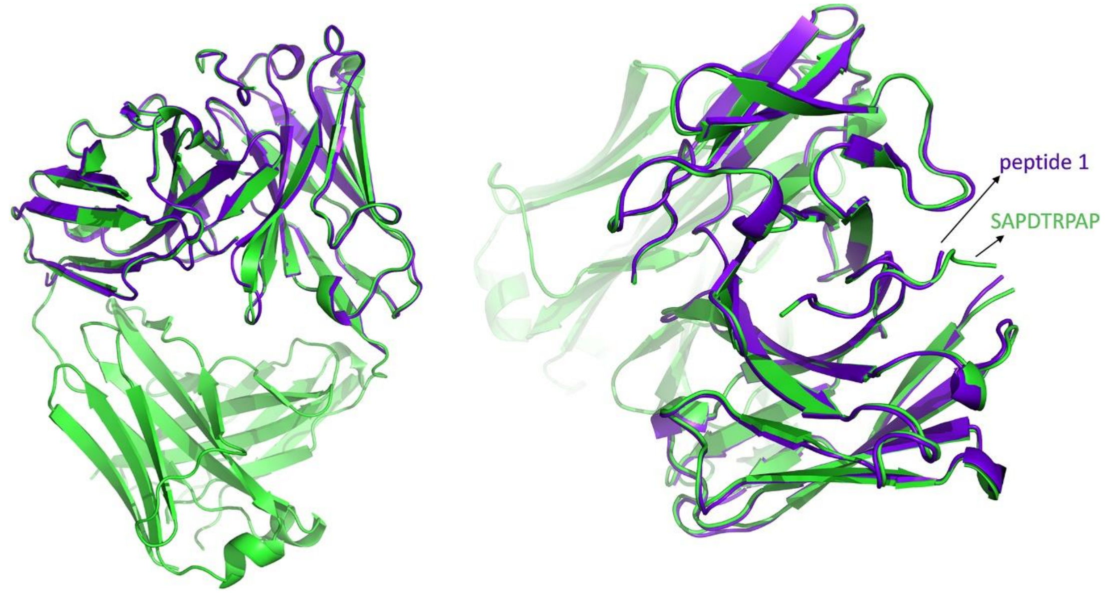

Figure S5. Superposition of SM3 Fab fragment (in green) in complex with a nonapeptide[S16] and the scFv-SM3 complexed to peptide **1** (this work, in purple).

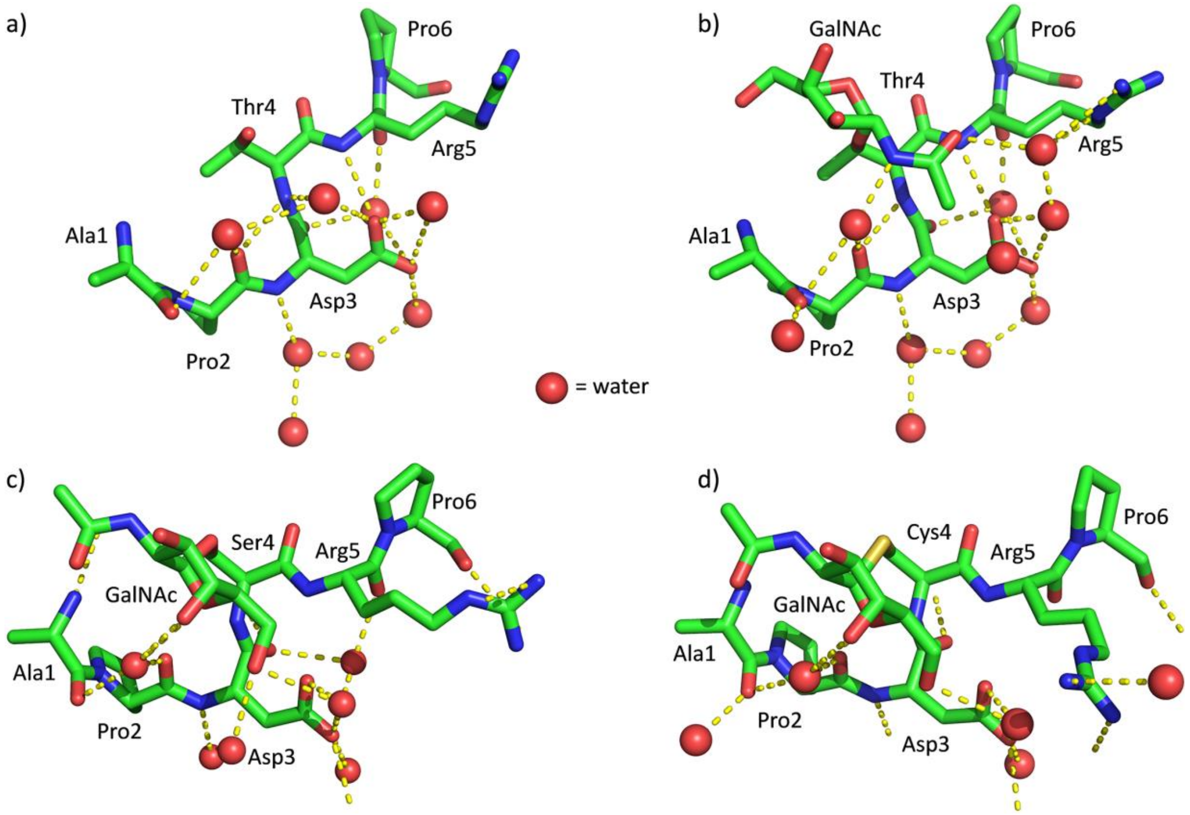

Figure S6. Hydrogen-bond network including water molecules for peptide **1** (a) and glycopeptides **1*** (b) and **2*** (c) and **3*** (d) in complex with scFv-SM3 obtained from the X-ray structures.

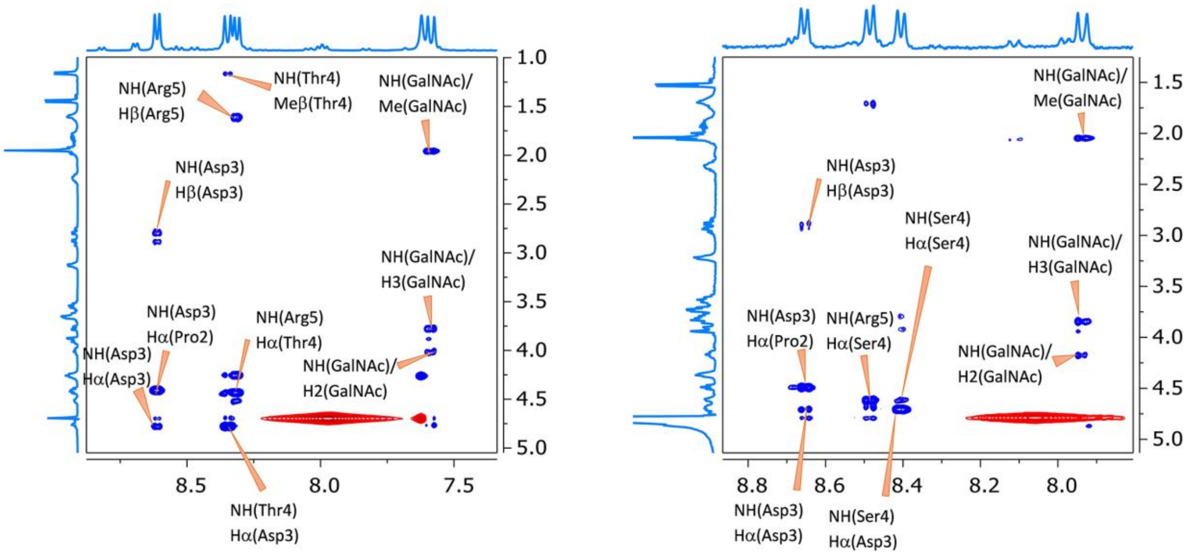

Figure S7. ROESY of glycopeptides **1*** (left panel) and **2*** (right panel) performed in H_2O/D_2O (9:1) at 298 K and pH=6.5.

Tables S6. Comparison of the experimental and MD-tar derived distances for glycopeptides **1*** and **2***. The experimental distances were semi-quantitatively determined by integrating the volume of the corresponding cross-peaks. All the distances are given in Å.

compound **1***

distances	Experimental values	DM_{H2O}-tar
NHArg5-HαThr4	2.1	2.2
NHThr4-HαAsp3	2.1	2.2
NHAsp3-HαAsp3	2.7	2.9
NHAsp3-HαPro2	2.1	2.2
NHThr4-NHGalNAc	3.1	3.1

compound **2***

distances	Experimental values	DM_{H2O}-tar
NHArg5-HαArg5	2.7	2.9
NHArg5-HαSer4	2.2	2.2
NHSer4-HαSer3	2.8	2.9
NHSer4-HαAsp3	2.2	2.2
NHAsp3-HαAsp3	2.7	2.9
NHAsp3-HαPro2	2.1	2.2
NHSer4-NHGalNAc	not observed	3.4

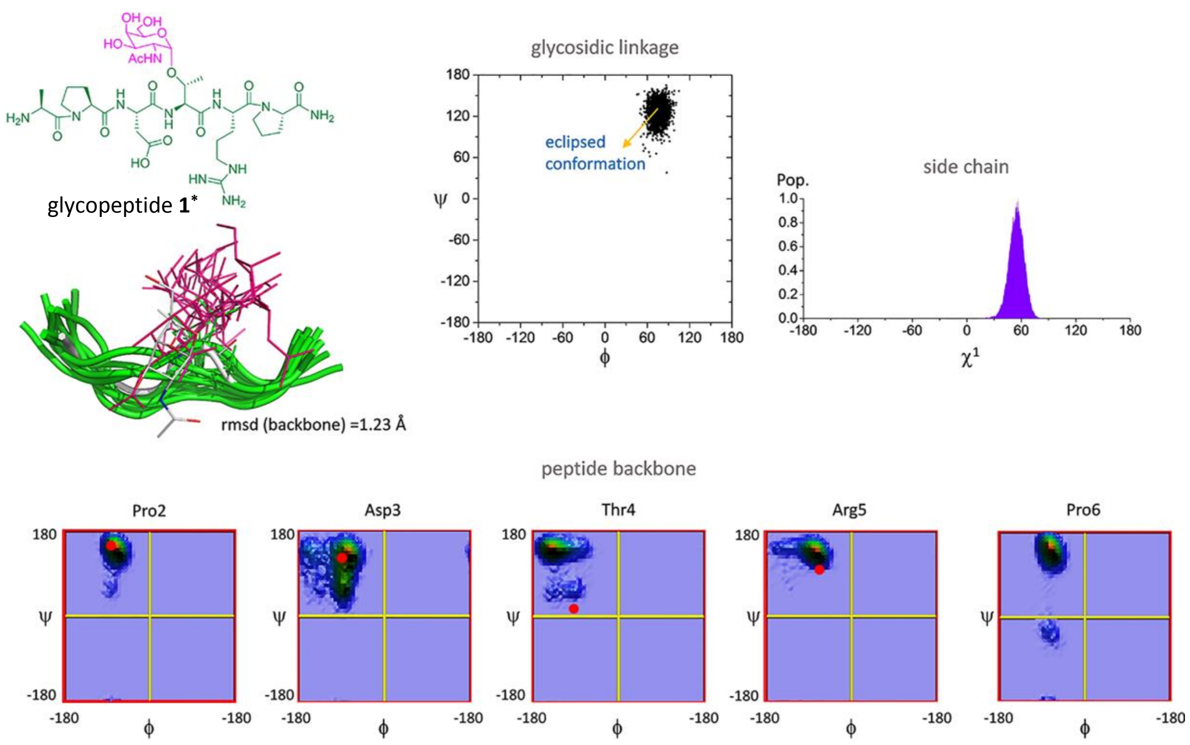

Figure S8. Ensembles obtained from 20 ns MD-tar simulations in explicit water performed on compound **1***, together with the χ^1 distribution for Thr4 and ϕ/ψ distributions for the peptide backbone and the glycosidic linkages. The peptide backbone is shown as a green ribbon and the sugar moiety is shown in pink. The conformation found for glycopeptide **1*** in the crystal structure in shown in grey. The glycosidic linkage displays mainly the typical eclipsed conformation.[S17] The ϕ/ψ values displayed for the backbone of glycopeptide **1*** in the complex with SM3 are shown as red dots.

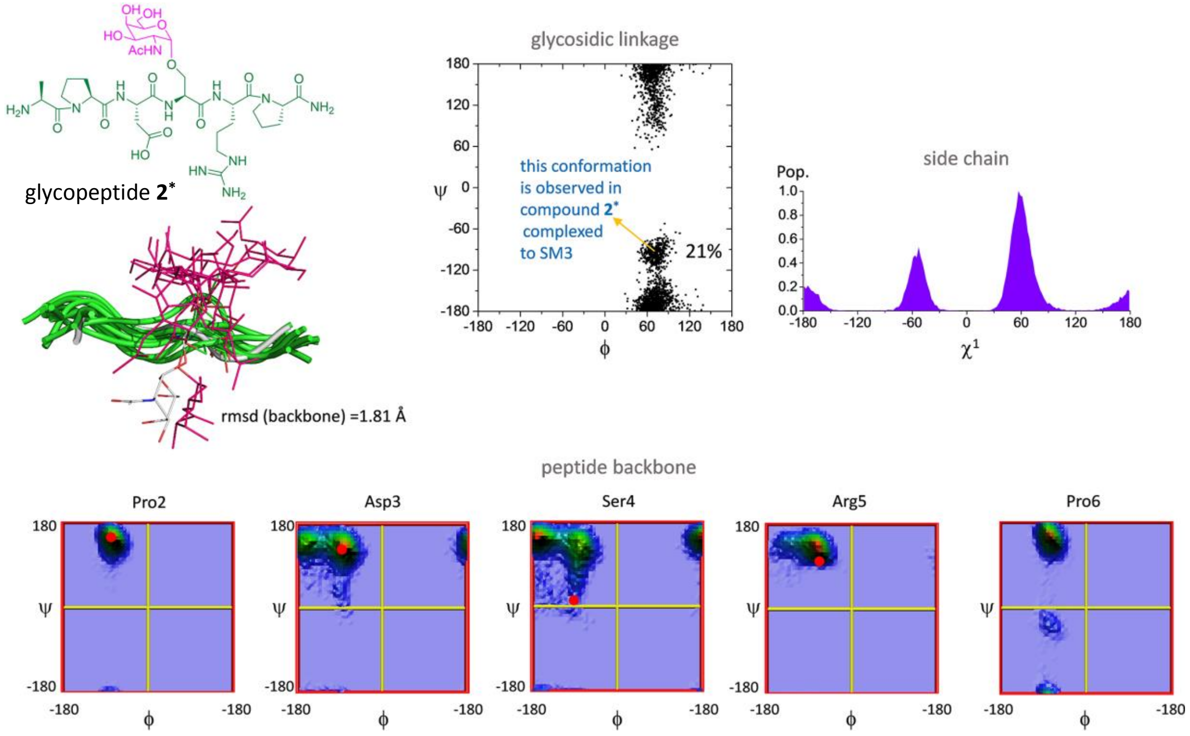

Figure S9. Ensembles obtained from 20 ns MD-tar simulations in explicit water performed on compound **2***, together with the χ^1 distribution for Ser4 and ϕ/ψ distributions for the peptide backbone and the glycosidic linkages. The peptide backbone is shown as a green ribbon and the sugar moiety is shown in pink. The conformation found in the crystal structure in shown in grey. The glycosidic linkage displays mainly the typical alternate conformation.[S18] Interestingly, a low population (21%) of the conformation observed in the X-ray structure of SM3:**2*** is also present through the MD simulations. The ϕ/ψ values displayed for the backbone of glycopeptide **2*** in the complex with SM3 are shown as red dots.

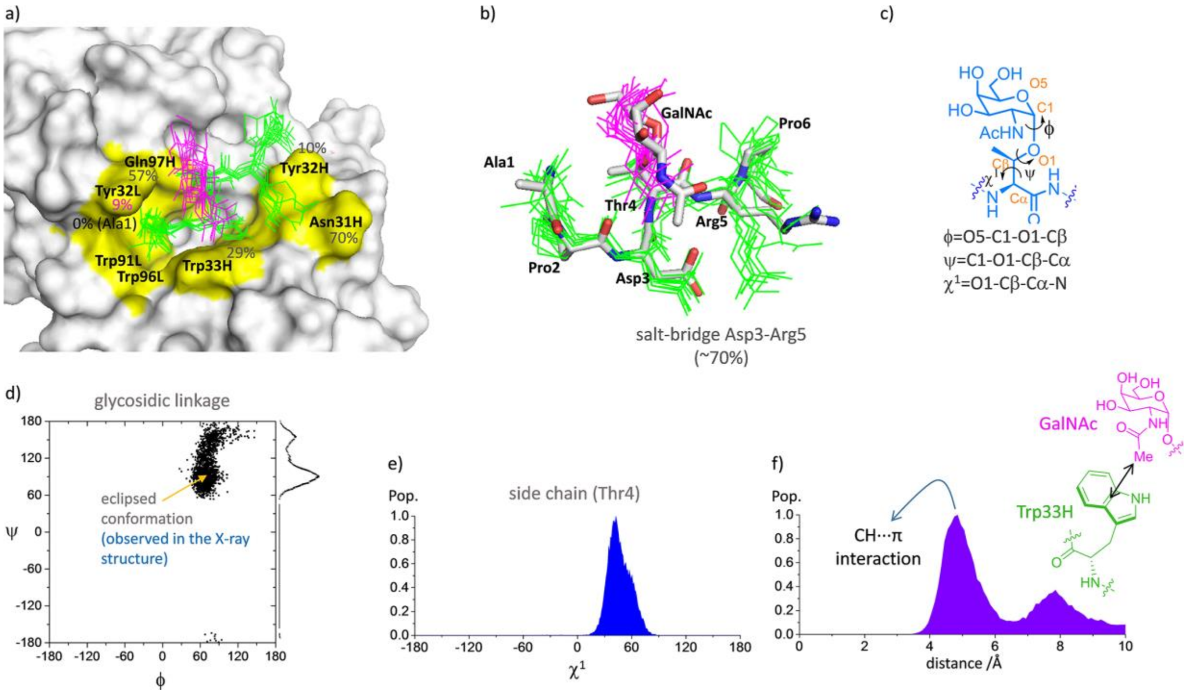

Figure S10. (a) Ensembles obtained from 20 ns MD simulations performed on scFv-SM3:**1***
complex in explicit water. The peptide is shown in green and the GalNAc unit in pink. Carbon atoms of key residues of SM3 antibody are in yellow. The population of the hydrogen bonds between glycopeptide **1*** and the antibody is indicated in grey (see main text). (b) Superposition of the ensembles obtained from the MD simulations and the conformation of compound **1*** in the crystal structure. (c) Definition of some significant torsional angles for the conformational analysis. (d) ϕ/ψ distribution for the glycosidic linkage through the MD simulations. (e) χ^1 distribution for residue Thr4 through the MD simulations. (f) Distance distribution between the methyl group of GalNAc and the centre of the aromatic ring of Trp33H. The MD simulations clearly indicate the existence of hydrophobic contact between GalNAc and the antibody.

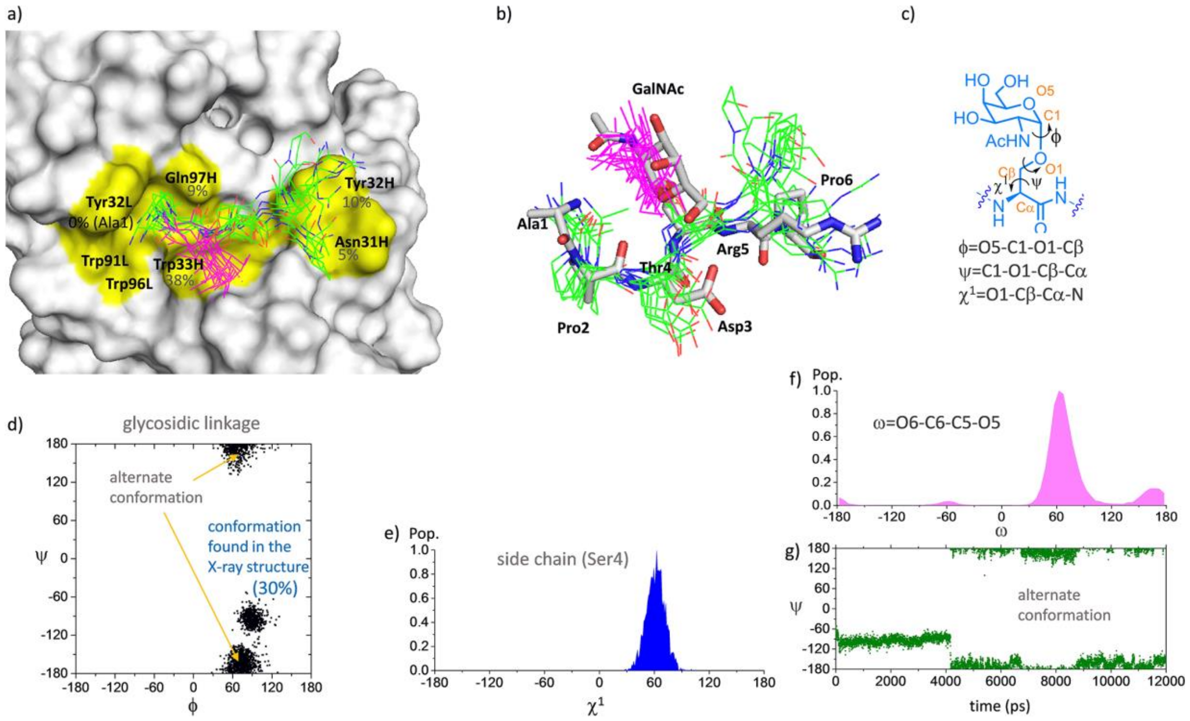

Figure S11. (a) Ensembles obtained from 20 ns MD simulations performed on scFv-SM3:**2*** complex in explicit water. The peptide is shown in green and the GalNAc unit in pink. Carbon atoms of key residues of SM3 antibody are in yellow. The population of the hydrogen bonds between glycopeptide **2*** and the antibody is indicated in grey (see main text). (b) Superposition of the ensembles obtained from the MD simulations and the conformation of compound **2*** in the crystal structure. (c) Definition of some significant torsional angles for the conformational analysis. (d) ϕ/ψ distribution for the glycosidic linkage through the MD simulations. (e) χ^1 distribution for residue Ser 4 through the MD simulations. (f) ω distribution (orientation of the hydroxymethyl group of GalNAc) through the MD simulations. (g) Time-series monitoring of the ψ torsional angle of the glycosidic linakge of compound **2*** in complex with scFv-SM3. The conformation found in the crystal structure is retained for the first 4 ns of the MD simulations.

References

[S1] C. Plattner, M. Höfener,; N. Sewald, *Org. Lett.* **2011**, *13*, 545.

[S2] D. Madariaga, N. Martínez-Sáez, V.J. Somovilla, L. García-García, M. A. Berbis, J. Valero-Gónzalez, S. Martín-Santamaría, R. Hurtado-Guerrero, J. L. Asensio, J. Jiménez-Barbero, A. Avenoza, J. H. Busto, F. Corzana, J. M. Peregrina, *Chem. Eur. J.* **2014**, *20*, 12616.

[S3] W. Kabsch, *Acta Crystallogr. D Biol. Crystallogr.* **2010**, *66*, 125.

[S4] M. D. Winn, C. C. Ballard, K. D. Cowtan, E. J. Dodson, P. Emsley, P. R. Evans, R. M. Keegan, E. B. Krissinel, A. G. Leslie, A. McCoy, S. J. McNicholas, G. N. Murshudov, N. S. Pannu, E. A. Potterton, H. R. Powell, R. J. Read, A. A. Vagin, K. S. Wilson, *Acta Crystallogr. D Biol. Crystallogr.* **2011**, *67*, 235.

[S5] P. Emsley, K. Cowtan, *Acta Crystallogr. D Biol. Crystallogr.* **2004**, *60*, 2126.

[S6] G. N. Murshudov, P. Skubak, A. A. Lebedev, N. S. Pannu, R. A. Steiner, R. A. Nicholls, M. D. Winn, F. Long, A. A.Vagin, *Acta Crystallogr. D Biol. Crystallogr.* **2011**, *67*, 355.

[S7] R. A. Laskowski, M. W. MacArthur, D. S. Moss, J. M. Thornton, *J. Appl. Cryst.* **1993**, *26*, 283.

[S8] W. L. Jorgensen, J. Chandrasekhar, J. D. Madura, R. W. Impey, M. L. Klein, *J. Chem. Phys.* 1983, *79*, 926.

[S9] D.A. Case, T.A. Darden, T.E. Cheatham, III, C.L. Simmerling, J. Wang, R.E. Duke, R. Luo, R.C. Walker, W. Zhang, K.M. Merz, B. Roberts, S. Hayik, A. Roitberg, G. Seabra, J. Swails, A.W. Götz, I. Kolossváry, K.F. Wong, F. Paesani, J. Vanicek, R.M. Wolf, J. Liu, X. Wu, S.R. Brozell, T. Steinbrecher, H. Gohlke, Q. Cai, X. Ye, J. Wang, M.-J. Hsieh, G. Cui, D.R. Roe, D.H. Mathews, M.G. Seetin, R. Salomon-Ferrer, C. Sagui, V. Babin, T. Luchko, S. Gusarov, A. Kovalenko, and P.A. Kollman (2012), AMBER 12, University of California, San Francisco.

[S10] V. Hornak, R. Abel, A. Okur, B. Strockbine, A. Roitberg, C. Simmerling, *Proteins: Struct., Funct., Bioinf.* **2006**, *65*, 712.

[S11] K. N. Kirschner, A. B. Yongye, S. M. Tschampel, J. González-Outeiriño, C. R. Daniels, B. L. Foley, R. J. Woods, *J. Comput. Chem.* **2008**, *29*, 622.

[S12] T. A. Andrea, W. C. Swope, H. C. Andersen, *J. Chem. Phys.* **1983**, *79*, 4576.

[S13] T. Darden, D. York, L. Pedersen, *J. Chem. Phys.* **1993,** *98*, 10089.

[S14] S. Dziadek, C. Griesinger, H. Kunz, U. M. Reinscheid, *Chem. Eur. J.* **2006**, *12*, 4981.

[S15] U. Karsten, N. Serttas, H. Paulsen, A. Danielczyk, S. Goletz, S. *Glycobiology* **2004**, *14*, 681.

[S16] P. Dokurno, P. A. Bates, H. A. Band, L. M. Stewart, J. M. Lally, J. M. Burchell, J. Taylor-Papadimitriou, D. Snary, M. J. Sternberg, P. S. Freemont, *J. Mol. Biol.* **1998**, *284*, 713.

[S17] F. Corzana, J. H. Busto, G. Jiménez-Osés, M. García de Luis, J. L. Asensio, J. Jiménez-Barbero, J. M. Peregrina, A. Avenoza, *J. Am. Chem. Soc.* **2007**, *129*, 9458.

[S18] F. Corzana, J. H. Busto, G. Jiménez-Osés, J. L. Asensio, J. Jiménez-Barbero, J. M. Peregrina, A. Avenoza, *J. Am. Chem. Soc.* **2006**, *128*, 14640.
